# Probabilistic Techno-Economic Assessment of Medium-Scale Photoelectrochemical Fuel Generation Plants

**DOI:** 10.1021/acs.energyfuels.4c00936

**Published:** 2024-06-22

**Authors:** Alexandre Cattry, Hannah Johnson, Despoina Chatzikiriakou, Sophia Haussener

**Affiliations:** † École Polytechnique Fédérale de Lausanne (EPFL), Institute of Mechanical Engineering, LRESE, 1015 Lausanne, Switzerland; ‡ Materials Engineering, Toyota Motor Europe NV/SA, Hoge Wei 33, 1930 Zaventem, Belgium

## Abstract

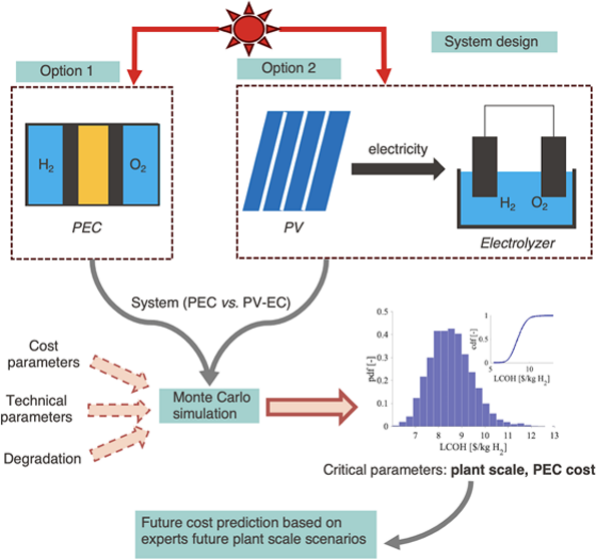

Photoelectrochemical (PEC) systems are promising approaches for sustainable fuel processing. PEC devices, like conventional photovoltaic-electrolyzer (PV-EC) systems, utilize solar energy for splitting water into hydrogen and oxygen. Contrary to PV-EC systems, PEC devices integrate the photoabsorber, the ionic membrane, and the catalysts into a single reactor. This integration of elements potentially makes PEC systems simpler in design, increases efficiency, offers a cost advantage, and allows for implementation with higher flexibility in use. We present a detailed techno-economic evaluation of PEC systems with three different device designs. We combine a system-level techno-economic analysis based on physical performance models (including degradation) with stochastic methods for uncertainty assessments, also considering the use of PV and EC learning curves for future cost scenarios. For hydrogen, we assess different PEC device design options (utilizing liquid or water vapor as reactant) and compare them to conventional PV-EC systems (anion or cation exchange). We show that in the current scenario, PEC systems (with a levelized cost of hydrogen of 6.32 $/kg_H_2__) located in southern Spain are not yet competitive, operating at 64% higher costs than the PV-driven anion exchange EC systems. Our analysis indicates that PEC plants’ material and size are the most significant factors affecting hydrogen costs. PEC designs operating with water vapor are the most economical designs, with the potential to cost about 10% less than PV-EC systems and to reach a 2 $/kg_H_2__ target by 2040. If a sunlight concentrator is incorporated, the PEC-produced hydrogen cost is significantly lower (3.59 $/kg_H_2__ in the current scenario). Versions of the concentrated PEC system that incorporate reversible operation and CO_2_ reduction indicate a levelized cost of storage of 0.2803 $/kWh for the former and a levelized cost of CO of 0.546 $/kg_CO_ for the latter. These findings demonstrate the competitiveness and viability of (concentrated) PEC systems and their versatile use cases. Our study shows the potential of PEC devices and systems for hydrogen production (current and future potential), storage applications, and CO production, thereby highlighting the importance of sustainable and cost-effective design considerations for future advancements in technology development in this field.

## 1 Introduction

Solar energy has an unlimited potential to sustain our energy demand. Covering just 0.25% of the earth’s surface with 10% efficient solar systems can meet the expected energy demand in 2050.^1^ However, solar energy is intermittent (e.g., on cloudy days), dilute (low power density), and unequally distributed (e.g., polar versus equatorial regions). One needs to store solar energy as a dense chemical fuel to overcome such shortcomings and to balance energy supply with demand. Among such chemical fuels, hydrogen offers two advantages: (i) the highest possible energy density per unit of mass (120 MJ/kg), and (ii) CO_2_-free and sustainable energy production. The European Union and the U.S. Department of Energy (DOE) consider these hydrogen fuel devices essential for the transition to green energy and a low-carbon economy.^2,3^ Moreover, solar energy can be harnessed to convert CO_2_ and H_2_O molecules into syngas.^4^ This approach can contribute to a reduction in anthropogenic CO_2_ emissions, transforming CO_2_ into a valuable feedstock. Solar chemicals, when stored (e.g., in compressed or liquid form^5^), can be transported from areas with abundant production (e.g., high solar irradiation regions) to locations with lower sunlight exposure. Additionally, these chemicals can be stored seasonally, transitioning from periods of high production (i.e., high solar irradiation) to times of reduced production (i.e., low solar irradiation). During lower production periods, the stored chemical energy can be subsequently converted into usable power and other energy vectors. Nonetheless, the optimal design for solar chemical fuel devices to produce energy at a large scale is unclear.

One essential part of such systems is the solar fuel reactor, where water or CO_2_ is converted into oxygen and hydrogen or CO, respectively. Here, we will focus on photodriven solar fuel approaches and not thermally driven approaches (such as solar thermochemical redox cycles). Two such photodriven technologies for hydrogen generation are (i) photovoltaic modules coupled to electrolyzer (PV-EC) systems and (ii) photoelectrochemical (PEC) devices. PEC approaches show a lower maturity. Both technologies can also be used to reduce CO_2_. Here, PEC devices refer to water (CO_2_) conversion reactors where the photoabsorber and the membrane-separated electrocatalyst are integrated into one device. Detailed knowledge of both performance and the techno-economic viability of each approach is required to evaluate and compare these systems in terms of efficiency or production rates. There are previous studies reporting on and comparing these solar hydrogen reactors (see, e.g., refs (6−9)). Here, however, we focus on the uncertainty of such techno-economic analyses, pay specific attention to the details of the reactor designs and how it can affect the cost, extend the analysis to different variations of PEC systems (operated with concentrated light, reversibly, or with CO_2_ as a reactant), and provide predictions of future costs following different installation and learning scenarios.

The PV-EC systems, a more conventional approach than PEC devices, have benefited from the independent optimization of their PV (where light is converted into electron–hole pairs) and EC (where charge carriers are transported and used to sustain an electrochemical reaction) components. However, the inclusion of wiring to interconnect the EC with PV arrays, along with the introduction of power electronics (such as DC–DC converters), introduces supplementary electrical, fluidic, and thermal losses. PEC devices, in contrast, circumvent these losses by employing photoelectrodes capable of simultaneously converting solar energy into electron–hole pairs and sustaining electrochemical reactions, all in a single, compact reactor. The balance of the system is also simplified as power electronics are not utilized, and less and much shorter electrical wirings are required (i.e., the wirings connecting the PV to the EC are not needed or consist of μm-scale connections). The development of PEC devices was marked by the initial achievement of water splitting with visible light, employing an n-type photoanode TiO_2_ and a platinum black cathode in 1972.^10^ Subsequently, diverse device designs were invented, which can be classified discretely from monolithic PEC configurations to integrated PEC (IPEC) devices^11,12^ (Figure S24, Discussion S15, and Table S3). Overcoming the stability challenge intrinsic to devices featuring a photoelectrode–electrolyte junction has posed considerable difficulties. The stability of these designs, defined by the ability to produce hydrogen consistently at a constant current density and efficiency, is often still less than 1 day,^13^ therefore likely requiring frequent replacement of the PEC components.

One of the first techno-economic analyses of PEC systems compared photocatalytic and PEC water splitting in four different systems.^6,14^ All systems showed practical challenges either due to safety concerns (cogeneration of an explosive mixture of oxygen and hydrogen in low-cost reactors) or high costs. Rodriguez et al.^15^ explored the effect of material choice on hydrogen price at the device level. They suggested that a relatively low production cost (<2.9 $/kg H_2_) was achievable if the PEC device design was improved in terms of geometry and material choice. Dumortier et al.^8^ showed the importance of integrating degradation parameters in techno-economic evaluations of PEC device designs where PEC replacement time was critical for the system’s efficiency. Their study evaluated a combination of catalyst and photoabsorber materials, minimizing hydrogen cost. Studies that combine design-dependent degradation rates and design-dependent balance of plants (BOP) costs in techno-economic evaluation are still limited.

Understanding the economics of PEC systems and their comparison to PV-EC systems is essential to guiding technology development and transfer. However, such studies are difficult, and fundamental questions (such as the impact of degradation specific to the device design and operating conditions) are answered with high uncertainty. In particular, it is not yet clear which design elements of the PEC device need to be improved so that PEC devices may offer an economic advantage over PV-EC systems. Shaner et al.^16^ evaluated PEC systems and predicted them at a marginally lower cost than the PV-EC system (11.4 vs 12.1 $/kg H_2_). Their study assumed that the EC stack replacements were necessary every 7 years, corresponding to ∼60,000 h in constant-use scenarios. The study carried out by Grimm et al.^7^ indicated, in contrast, that utilizing PEC approaches was more expensive than PV-EC technologies and showed the significant uncertainties associated with its practical implementation. Such uncertainties can be addressed with a stochastic approach, as has been done in a few previous studies when considering probabilistic hydrogen production costs for PV-EC systems^17^ or predicting the future price of hydrogen produced by proton exchange membrane ECs (PEMECs) and by gasifiers in 2030 and 2050.^18^

Utilizing an optical concentrator to enhance light concentration and thereby increase the photocurrent density and open-circuit voltage of the photoabsorber represents a theoretically favorable approach for improved performance and reduced costs.^6,8^ Concentrated systems introduce an additional variable (degree of freedom) of optimization, which is the relative geometrical area between the optical concentrator and the photoabsorber. Achieving an irradiation concentration of 1000 allows for a significant reduction in the photoabsorber area (by about a factor of 1000), making it economically feasible to adopt efficient and expensive III–V materials as photoabsorbers. Recent advancements have demonstrated the possibility of attaining average irradiation concentrations above 800 for scaled hydrogen production.^19^ The integrated PEC device demonstrated by Tembhurne et al.^20^ for water splitting and by Boutin et al.^26^ for CO_2_ reduction lies between a monolithic PEC device and the PV-EC according to Jacobsson’s design concepts,^11^ and is well-suited for utilizing a concentrator. In such a design, water (the reactant) flows in a channel contained between a quartz glass and the photoabsorber. The water absorbs the infrared part of the light, and by controlling its flow rate, the photoabsorber is cooled by convection. By controlling the temperature increase in the photoabsorber, the photoabsorber operates more efficiently. The preheated water then flows in the anode’s EC part of the device.

In addition to hydrogen production, exploring storage applications with PEC devices can be considered by employing reversible PEC operation. This involves operating the PEC in photodriven electrolysis mode to produce hydrogen during periods of sunlight and subsequently switching to fuel cell mode for power generation during high energy demand, similar to the operational principle of unitized regenerative fuel cells (URFCs), which operate in the dark (grid-connected) to produce hydrogen and then reuse it for power generation. Recent developments on URFCs have demonstrated the feasibility of achieving industrial-scale current densities (1 A/cm^2^) at a cost of 0.308 $/kWh,^21^ making it competitive with other storage technologies such as pumped-hydro, lithium-ion, flywheel, and vanadium redox-flow systems (0.15–0.8 $/kWh).^22^ However, the reversible operation of concentrated PEC devices utilizing photogenerated current density for hydrogen production has only just been demonstrated on the lab scale,^23^ and its economic competitiveness needs to be assessed.

Furthermore, PEC devices have the potential to contribute to the decarbonization of chemical production processes (e.g., of hydrocarbons), which are currently dominated by high-temperature thermochemical methods utilizing fossil fuels. Low-temperature CO_2_ electrosynthesis, driven by grid electricity, holds promise for cost-competitive CO production at 0.44 $/kg_CO_^24,25^ (compared to the market price of 0.6 $/kg_CO_). Recent experimental demonstrations of concentrated photoelectrochemical reduction of CO_2_ also show potential for cost-efficient CO production using concentrated light.^26^ However, while techno-economic evaluation of photodriven electrolysis utilizing nonconcentrated light and silicon photoabsorber has been performed with a high cost of 10.94 $/kg_CO_,^27^ there is currently no techno-economic study evaluating technical and economic competitiveness for concentrated PEC CO_2_ reduction.

To move toward designs that consider both technical and economic aspects of water-splitting methods, we consider three standard PEC devices (including one operating with water vapor) and compare them against two typical PV-EC devices in terms of systematic and physical performance models (including degradation rates). We combine this information with economic analysis to form a comprehensive techno-economic evaluation. Then, we consider technical and economic uncertainties using a stochastic (Monte Carlo) model while integrating essential elements of PEC designs (such as degradation rates). Additionally, we consider design performance under hypothetical future hydrogen cost scenarios. Such scenarios are defined by utilizing the learning rates of various systems and their estimated future installed cumulative capacity.^28^ Eventually, we evaluate the cost-effectiveness of employing PEC devices operated with concentrated light or operated reversibly (i.e., they can switch between photodriven electrolysis and fuel cell operation). Furthermore, we assess the cost-effectiveness of utilizing PEC devices for the reduction of CO_2_.

## 2 Methodology and Governing Equations

### 2.1 Solar Fuel System Description

**Figure 1:**
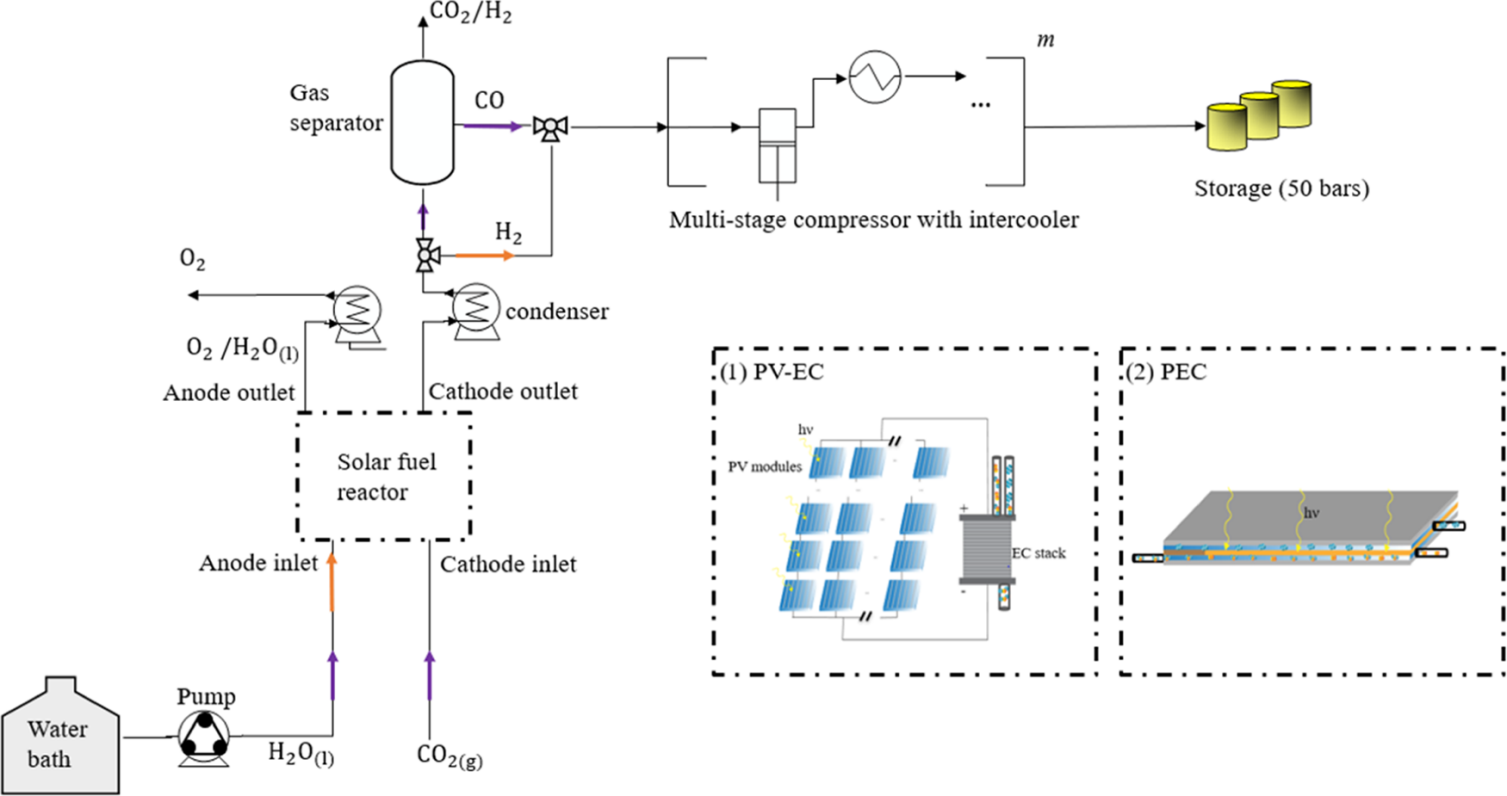
Schematic of a solar fuel system using two solar fuel reactors: (1) PV-EC system and (2) PEC device. The orange and purple arrows correspond to the plant operation for water-splitting reaction or CO_2_ reduction.

Electrolysis of water by two competing solar fuel reactors is considered: (1) PV-EC systems and (2) PEC systems. The two medium-sized systems, sized to produce on average 1000 kg of solar fuel (CO or H_2_) per day over 20 years and assuming 8 operating hours per day, are sketched in Figure 1. Assuming a 0.55 kg_H_2__/100 km consumption for hydrogen-fueled cars, ∼2000 cars driving 100 km per day can be recharged by such a system. A large-scale plant, as analyzed by Shaner et al., would correspond to at least 1 order of magnitude more, i.e., more than 10 t_H_2__/day. In system 1, feedwater pumps supply liquid water to the water-splitting EC stack. The pumps compensate for pressure drop in the pipes and pressurize water to the EC stack’s operating pressure. The EC stack is directly coupled to PV modules, which convert solar energy into electrical power. The number of PV modules in parallel and series is designed to ensure that the EC stack operates at its nominal current. The number of cells in the EC stack is optimized according to the system’s daily fuel production rate. Electrolysis of water results from the two half-reactions
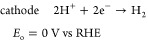
1
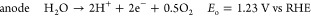
2

Oxygen produced on the anode side is separated from water and vented at the system’s outlet. Hydrogen is collected at the cathode outlet and subsequently separated from water. Before injecting hydrogen into the pipelines (at 50 bar), hydrogen is pressurized in a piston-type compressor, where a maximum compression ratio of π_max_ = 5.5:1 is achievable.^16^ A single compressor is sufficient for an EC stack operating at 20 bar. System 2 is similar to system 1, except for the solar hydrogen reactor. Light absorption and electrolysis of water happen in the same device. Photogenerated electron–hole pairs in the photoabsorber are separated and collected at the cathode and anode. PEC devices are assumed to operate at ambient pressure, which implies the requirement of a multistage compressor. The number of necessary compression stages *m* is given by the upper integer value of ln(*P*^out^/*P*^cathode^)/ln(π_max)_, where *P*^out^ is the gas network pressure. To minimize the compression work, intercoolers are placed between each compressor stage.

PEC devices are not restricted solely to hydrogen production. There are PEC device designs that can efficiently operate with gas on the cathode side for the reduction of gaseous CO_2_ into CO (i.e., gas diffusion electrode-based designs). Other PEC device configurations requiring a liquid electrolyte can be operated with a bicarbonate electrolyte^29^ where gaseous CO_2_ is dissolved in a KOH solution. In this study, gaseous CO_2_ is captured from a specific point source and introduced into the cathode side of the PEC device. Here, it undergoes reduction into CO. To prevent membrane dehydration,^30^ humid CO_2_ is utilized. This presence of water also facilitates the conversion of water into H_2_ and can potentially limit the Faradaic efficiency for CO. The electro-reduction of CO_2_ with concurrent hydrogen production is governed by the following half-reactions
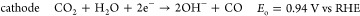
3
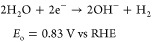
4
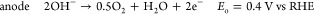
5implemented, for example, in a zero-gap gas-fed anion exchange EC operated with liquid water on the anode.^31^

We use southern Spain, i.e., Sevilla, as a reference location, where the annual global horizontal irradiation (GHI) is 1840 kWh/m^2^. The yearly averaged GHI is 630 W/m^2^ over eight operational hours (i.e., 1840 kWh/(8 h × 365 days)).^32^ If an optical concentrator is utilized, the plant would be installed north, e.g., Castello, to have the direct normal irradiation (DNI) equal to the GHI in Sevilla, therefore comparing concentrated and nonconcentrated devices with the same energy input.

The energy consumption associated with gas separators is not considered in the context of water splitting since hydrogen generation occurs on the cathode side and is effectively separated from the reactant introduced on the anode side. A condenser is necessary to separate hydrogen from water vapor, but its cost is minimal compared to other utility expenses.^6^ However, in the case of gaseous CO_2_ reduction, gas separators are indispensable. This is because the cathodic stream contains a gas mixture of CO_2_, CO, H_2_, and water vapor. Achieving high CO_2_ conversion efficiency is often challenging, with efficiency values typically remaining below 45%.^33^ Furthermore, a Faradaic efficiency of at least 5%^25^ is allocated to hydrogen, necessitating the use of these separators. Following the gas separation process, CO is pressurized to 50 bar.

Hydrogen storage is complex, and its cost is not evaluated in the system’s boundary, even though its cost is not negligible (375 €/kg_H_2__ at 50 bar^34^). An alternative to compressed hydrogen storage is the use of hydrogen for ammonia synthesis alongside N_2_ fixation.

For simplicity, only the degradation of the solar fuel device is considered. Other plant components operate at constant efficiency.

Figure 1 shows a simplified sketch of the system’s BOP. The BOP is specific to the PV-EC and PEC design. The BOP differs in the number of separators, dryers, valves, tanks, compressors, pumps, heat exchangers, and pipes utilized in the system. This study explicitly computes the balance of plant cost and physical performance of each PEC system and each PV-EC system (with two different EC types each) modeled.

### 2.2 Performance Model

The performance of a solar fuel reactor is evaluated by the solar-to-fuel (STF) efficiency. The yearly averaged STF efficiency is calculated based on the Gibbs free energy (*G*_0_) under standard temperature and pressure conditions, which is typically used in the photoelectrochemistry community^36^
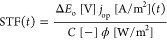
6where *j*_op_(*t*) is the yearly averaged operating current density (per photoabsorber area), Δ*E*_0_ is the thermodynamic potential difference between two half-reactions (Δ*G*_0_ = −2*F*_a_Δ*E*_0_), ϕ is the averaged global horizontal irradiation, and *C* is the optical concentration. The STF efficiency averaged over the system lifetime is
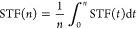
7where *n* is the plant’s lifetime. The hydrogen production rate *ṁ*_H_2__ is directly derived from the operating current density by using Faraday’s law. For CO, the production rate is
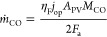
8where the Faradaic efficiency is assumed to be 95% (hydrogen coproduction accounts for 5%).

#### 2.2.1 PV-EC System

##### 2.2.1.1 Photovoltaic Module

The crystalline silicon (c-Si) PV module technology is the most mature technology among all commercial solar modules. By 2020, the cumulative PV module production reached 773 GW_p_.^37^ The c-Si PV modules, most relevant for terrestrial applications, are chosen for PV-EC systems. From the NREL database on module efficiency record,^38^ a 20.4% efficient c-Si PV module is selected. Table 1 shows the technical parameters relevant to the modeling equations.

**Table 1:** C-Si PV Module Technical Parameters at 25°C and AM1.5G (1000 W/m^2^)

parameters	value	unit	references
open-circuit voltage	0.665	[V]	(39)
short current density	397.37	[A/m^2^]	(39)
efficiency	20.4%	[%]	(38)
degradation rate	0.5	[%/year]	(40)
fill factor	0.772	[-]	(39)

The PV module is modeled as a nonideal diode in parallel with an ideal source current^41^
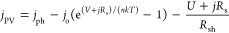
9where *j*_ph_ is the photocurrent density, approximated as the short current density *j*_sc_, *n* is the ideality factor, *j*_o_ is the diode reverse saturation current, *R*_s_ is the series resistance, and *R*_sh_ is the shunt resistance. The short current density and the open-circuit voltage are calculated with respect to reference conditions (25 °C and ϕ_0_ = 1000 W/m^2^) as^8^
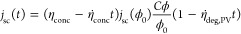
10
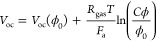
11where *R*_gas_ is the universal gas constant. The degradation rate η̇_deg,PV_ of the c-Si PV module is modeled as a decrease of the short current density over time.^40^ If an optical concentrator with an optical concentration ratio of *C* is utilized, the concentrator optical efficiency is η_conc_ and its degradation rate is η̇_conc_.

##### 2.2.1.2 Electrolyzer Stack

Most of the industrial-scale electrolyzers are alkaline (AEC) today. The AEC is cheaper than other electrolyzers and available for industrial-scale applications.^42^ However, dynamic operation with AEC is limited and, therefore, more challenging to operate with PV modules. A PEMEC system is more expensive due to its use of Nafion membranes and costly catalysts. However, PEMEC’s ability to rapidly respond to load change makes them particularly suitable for operating with intermittent energy sources.^43^ Technical parameters of PEMEC and AEC systems working in liquid water and relevant to our modeling equations are shown in Table 2.^44−47^

**Table 2:** AEC and PEMEC Technical Parameters

parameters	PEMEC	AEC	unit	references
operating temperature	60	70	[°C]	(45)
cathode pressure	20	20	[bar]	(45)
anodic exchange current density	0.011	0.08	[A/m^2^]	(44,46)
cathodic exchange current density	42	4	[A/m^2^]	(44,46)
anodic Tafel slope	47	86	[mV/dec]	(44,46)
cathodic Tafel slope	30	77	[mV/dec]	(44,46)
resistance	1.15 × 10^–5^	3.448 × 10^–5^	[Ω m^2^]	(44,46,47)
nominal operating current density	18,000	6000	[A/m^2^]	(45−47)
cell area	0.2	2	[m^2^]	(45)
degradation	10	2	[μV/h]	(45)

The operating voltage of the EC is the sum of the standard thermodynamic equilibrium potential *E*_0_ and different current-dependent overpotentials^8^
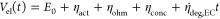
12

The different terms in eq 12 are defined in the Supporting Information (eqs S1–S8).

##### 2.2.1.3 PV-EC Coupling

A maximum power point tracker (MPPT) DC–DC converter is often used to operate the PV module at its maximum power point (MPP). This is at the expense of a conversion efficiency loss of 5% and an additional power electronic cost. Here, the MPPT DC–DC converter is discarded, i.e., the PV and the EC operate at the same current–voltage point (Figures S1 and S2). Operating the PV-EC system at a point close to the MPP can be achieved by designing an optimal number of PV modules in series and parallel. Like this, gas production yields similar to a PV-EC system coupled with a 95% MPPT DC–DC converter can be obtained.^48^ In this work, an optimal number of PV modules in series and parallel is calculated considering constant but design-specific degradation rates. Technical parameters are yearly averaged, and our study does not consider dynamic loads. This represents a hypothetical PV-EC system operating in constant solar irradiation and environmental conditions. A comparison with a system where PV and EC are connected via an MPPT is given in the results.

#### 2.2.2 PEC System

##### 2.2.2.1 Liquid Water Operation

**Figure 2:**
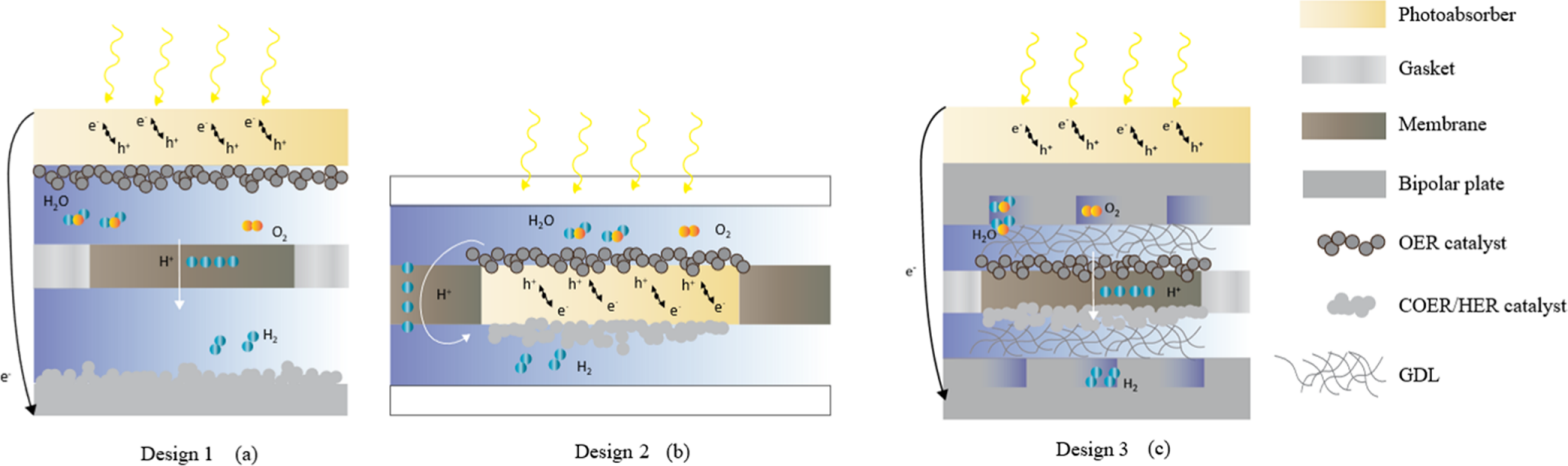
Three different PEC designs shown here for water splitting: (a) wired PEC device, (b) monolithic PEC device, and (c) integrated PEC device.

Three different PEC device designs, shown in Figure 2, are chosen. Design 1 is wired with the hydrogen evolution reaction (HER) (or the CO evolution reaction (COER) in the CO_2_ reduction case) and oxygen evolution reaction (OER) catalysts immersed in a liquid electrolyte and separated by a membrane and a gasket. A wire is employed to electrically connect the photoabsorber, which is coated with OER catalysts, to the dark cathode.^49^ Design 2 is wireless with the HER and OER catalysts coated on the photoabsorber and immersed in the electrolyte. The OER catalyst is coated on the photoabsorber’s face facing the incident light, and the HER catalyst is coated on the backside. A membrane physically separates the cathode from the anode and surrounds the photoabsorber to ensure ionic transport and prevent product gas mixture.^50^ Design 3 is wired with membrane-separated electrocatalysts closely integrated with the photoabsorber within the same reactor. A bipolar plate distributes the reacting flow and electrically connects the photoabsorber with the gas diffusion layer.^20^ For design 3, a version with a gaseous reactant (i.e., water vapor or carbon dioxide) is considered as well. Common to the three designs is the electron–hole pairs photogeneration in the photoabsorber. The holes and electrons are transported to the OER and HER/COER catalysts, respectively. The electrolyte and the membrane ensure ionic transport and product separation.

The membrane-separated electrocatalyst characteristics are taken from the PEMEC technology at ambient temperature. The photoabsorber is modeled assuming that it performs similarly to a triple junction a-Si/a-Si/μc-Si cell with an open-circuit voltage of 2.2 V and a short current density of 71 A/m^2^ (at 1000 W/m^2^ and 25 °C), performance characteristics desired for eventual photoelectrodes (likely a dual absorber). For design 3 (l), which can also operate with concentrated light, the photoabsorber is assumed to perform similarly to III–V material-based triple junction PV cells. The assumed open-circuit voltage and the short current density are 2.55 V and 146 A/m^2^ at 1000 W/m^2^ and 25 °C.

Linear degradation rates are estimated from values found in ref (51) and are reported in Table S1. For PEC design 3, it is assumed that the photoabsorber degradation is overestimated in ref (51) for an operating time above 30,000 h. For a photoabsorber not in contact with the electrolyte, degradation rates of a thin-film solar cell in an ambient environment are considered more realistic. The photoabsorber degradation is assumed to be 1%/year.^52^ As the thin-film solar cell degradation is mainly due to the open-circuit degradation,^40^ 0.75 and 0.25%/year degradation rates are assumed for the open-circuit voltage and the short current density, respectively. Table 3 summarizes the PEC technical parameters of the three designs. Different ohmic resistances are used for each design. Design 2 also differs from the other designs as the photoabsorber and the membrane area are exposed to irradiation, reducing the rate of electron–hole pairs that are photogenerated per unit of the total reactor area. The water/electrolyte on top of the semiconductor does not limit the transmittance of light to the semiconductor but instead increases it compared to designs 1 and 3 (see Figure S7). This results from the disparity in the real part of the refractive indices between water and the photoabsorber, which is narrower compared to air with the photoabsorber, resulting in reduced reflectivity (similar to an antireflection coating). Additionally, water predominantly absorbs light in the infrared spectrum while remaining transparent to ultraviolet (UV) and visible light, which is a useful part of the light spectrum for generating electron–hole pairs.

**Table 3:** PEC Technical Parameters

parameters	value	unit	references
operating temperature	20	[°C]	(8,51)
cathode pressure	1	[bar]	(8,51)
Membrane-Separated Electrocatalysts			
anodic exchange current density, IrO_2_	10^–3^	[A/m^2^]	(8,44,51)
cathodic exchange current density, Pt (HER)	10	[A/m^2^]	(8,44,51)
Ag (COER)	4.71 × 10^–3^		(53)
anodic Tafel slope, IrO_2_	47	[mV/dec]	(8,44,51)
cathodic Tafel slope, Pt (HER)	30	[mV/dec]	(8,44,51)
Ag (COER)	66		(53)
resistance*		[Ω m_pa_^2^]	
zero-gap solid electrolyte	1.27 × 10^–5^ (design 3)		(44)
membrane + electrolyte	4.57 × 10^–5^ (design 3 COER)		(54)
	1.53 × 10^–5^ (design 1)		(55,56)
	1.8 × 10^–5^ (design 2)		(55,56)
degradation	Table S1	[h^–1^]	(51)
Photoabsorber			
open-circuit voltage, 3-j Si	2.2	[V]	(15)
III–V	2.55		(8,44,20)
short current density, 3-j Si	71	[A/m^2^]	(15)
III–V	146		(8,44,20)
series resistance, 3-j Si	0.001	[Ω m^2^]	fit to^15^
III–V	0.0000001 (ideal)		(8)
shunt resistance, 3-j Si	0.5	[Ω m^2^]	fit to^15^
III–V	10000 (ideal)		(8)
degradation	Table S1	[h^–1^]	(51)
open-circuit voltage degradation, *V*_oc_	0.75 (design 3)	[%/year]	(40,52)
short current density degradation, *j*_sc_	0.25 (design 3)	[%/year]	(40,52)
Concentrator			
initial optical efficiency	85	[%]	(8)
concentrator degradation	0.65	[%/year]	(8)

For a reversibly operating device (i.e., electrolysis and fuel cell mode), the fuel cell current–voltage characteristic is calculated in analogy to the EC characteristic: *V*_fc_(*t*) = *E*_0_ – η_act_(*F*_c,_*t*) – η_ohm_(*F*_m,_*t*). The discharge/charge phase ratio is assumed as 2/3.^21^ Therefore, the FC operates for 5.333 h during the discharge phase after charging in photodriven EC mode for 8 h at the same current as the EC mode. The produced electrical power is *V*_fc_(*t*) × *I*_op_(*t*).

For the device conducting a CO_2_ reduction reaction, a silver catalyst is selected for its ability to selectively produce CO.^57^ To minimize the unwanted HER, an anion exchange membrane and alkaline conditions are preferred over Nafion and acidic conditions. However, when exposed to CO_2_, the OH^–^ ions are converted into carbonate/bicarbonate,^58^ which decreases the conductivity of the membrane. The silver cathodic overpotential is 4.71 × 10^–3^ A/m^2^ and the charge transfer coefficient is 0.44, resulting in a Tafel slope of 66 mV/dec.^53^ The ionic conductivity of a bicarbonate/carbonate 32 μm thick PiperION membrane is 0.75 S/m at 30 °C,^54^ resulting in a 4.57 × 10^–5^ Ω m^2^ resistance. Due to limited data on the long-term operation (>10,000 h) of low-temperature CO_2_ electrolyzers, it is challenging to estimate degradation rates. Therefore, it is assumed that the degradation rate is the same as that of a solid oxide CO_2_ electrolyzer, which has been proven to operate for over 7600 h at a degradation rate of 22 μV/h, even though it is very likely that the degradation rate is higher in current low-temperature electrolyzers.^59^ Based on recent experimental results from a zero-gap gas-fed-electrolyzer, it has been demonstrated that the CO_2_ electrolyzer can reach a current density of 470 mA/cm^2^. Therefore, a limiting current density of 500 mA/cm^2^ is assumed for the CO_2_ electrolyzer.^60^

The photoabsorber is modeled as a diode in parallel with an ideal source current (in the same fashion as for the PV)^41^
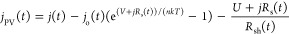
13

The photocurrent density, the series resistance, the shunt resistance, and the open-circuit voltage are prone to transient degradation. The polarization curve of the membrane-separated electrocatalysts is^8^
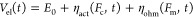
14

The activation overpotential and the ohmic overpotential increase over time due to degradation. Replacement of the PEC device resets the performances to initial conditions. The current-dependent overpotentials also depend on the current concentration factors *F*_c_ and *F*_m_, defined as the ratio between the photoabsorber (PA) and the catalyst projected area and the PA and the membrane area, respectively.

The ohmic resistance depends on the design. For design 1, the ohmic resistance is
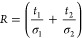
15where the membrane ionic conductivity is σ_1_ = 10 S/m, the electrolyte conductivity is σ_2_ = 38 S/m, the membrane thickness is *t*_1_ = 127 μm, and the height of the electrolyte channel is *t*_2_ = 1 mm.^55,56^

For design 2, the ohmic resistance is calculated as^56^
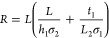
16where *L* = 0.1 cm is the length of the photoabsorber, *h*_1_ = 0.5 mm is the height of the channel (assumed less than 1 mm due to low operating current densities and low bubble formation rate), *t*_2_ is the thickness of the membrane, and *L*_2_ = 0.1*L*_1_ is the length of the membrane (the membrane area is 10 times less than that of the photoabsorber).

For design 3, the ohmic resistance is
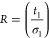
17

The operating point of the PEC device is deduced from the intersection of the photoabsorber and the membrane-separated electrocatalysts’ current–voltage curves (Figures S3–S6).

##### 2.2.2.2 Water Vapor Operation

PEC devices operating with water vapor are modeled with the same technical parameters used for the PEC devices operating with liquid water, except for the ionic resistance, the limiting current density, and the thermodynamic equilibrium potential. The ionic conductivity of the Nafion membrane at 20 °C and humidified with air being at a relative humidity (RH) of 80% is^55^
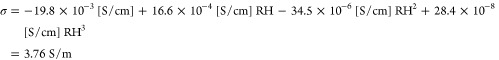
18

A constant RH = 80% is assumed in this study but can be increased to 100% if the air is bubbled through a water bath (the ionic conductivity would be 8.52 S/m for RH = 100%). The ionic resistance of design 3 operating with water vapor is 3.38 × 10^–4^ Ω m^2^. For design 2, the ionic resistance is calculated with design parameters found in ref (61). Two alternative reference scenarios are proposed for design 2. In the first design, considering a 2 μm thick Nafion layer on top of the photoabsorber, a 0.1 cm long photoelectrode, and a cell length of 0.1 cm, the resistance is 1.3 × 10^–1^ Ω m^2^, which would lead to an excessive ohmic drop (13 V at 10 mA/cm^2^). In the second design, the Nafion overlayer is 200 μm thick, and its length is more considerable than the photoabsorber. This would lead to a high cost for the PEC device. Therefore, design 2 operating with water vapor was discarded from this study. Design 1 would be operational with water vapor by finding a new photoabsorber structure. The photoabsorber would be porous, with catalysts deposited and embedded in an ionomer.^62^ Due to insufficient data regarding the costs of this manufacturing process at an industrial scale, design 1 operating with water vapor is also not considered.

The limiting current density depends on the design, operating RH, gas flow rate, and temperature. A limiting current density of 500 A/m^2^ is assumed.^63,64^ 500 A/m^2^ is greater than the short current density of the photoabsorber illuminated with a GHI of 630 W/m^2^ and designed with an area 10 times larger than the EC part (*F =* 10). In this case, the intersection of the PA and EC *IV* curves occurs when enough water is supplied to the membrane to keep it well hydrated. A limiting current density below 400 A/m^2^, i.e., in a water starvation regime, would make the PEC device more expensive than the liquid-based one (Figure S14). That is why, in order not to discard PEC design 3 (v) from this study, it is assumed that a hypothetical current density of up to 500 A/m^2^ can be achieved with operating conditions maintaining the membrane well hydrated.

### 2.3 Cost Model

The levelized cost of hydrogen is utilized to compare the economic viability of the PV-EC and PEC systems. It is defined as^7^
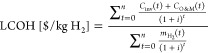
19where *C*_inv_ is the total investment cost, *C*_O&M_(*t*) is the yearly averaged operational cost at year *t*, *i* = 6% is the discount rate (taken from ref (17) and rounded up), and *m*_H_2__ is the yearly averaged hydrogen production at year *t*. The investment cost, as well as the operational and maintenance (O&M) cost, are specifically defined for the PV-EC and PEC systems below. To produce CO, *m*_CO_ replaces *m*_H_2__ in the LCOH formula, giving LCOCO. To generalize, we refer to LCO of fuel (LCOF).

The cost performance of the reversible PEC operation is assessed with the levelized cost of storage^22^
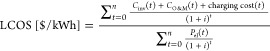
20

As no external electricity is supplied to the reversible PEC (the integrated PA supplies the photocurrent) for splitting water into hydrogen and oxygen, the charging cost = 0. *P*_el_ is the electricity produced during the discharge phase.

#### 2.3.1 PV-EC System

The cost of the photovoltaic system includes the photovoltaic module, the electrical cabling, the mounting of the PV module, and yearly operation and maintenance costs. The O&M costs are calculated with respect to the PV-EC system operating power point. If necessary, the EC stack is replaced after 10 years (in the middle of the plant’s lifetime). Considering an electrolyzer learning rate of 18% with a 0.36 GW/year capacity growth, the ratio of the EC stack cost in 2030 and now is 66%.^28,65^ A more conservative value of 70% is chosen. An operational and maintenance cost is associated with the EC stack replacement. The replacement cost of the electrolyzer accounts for 15% of the stack cost at the year of replacement.

The cost for power electronics is not considered, as the PV modules are directly coupled to the electrolyzer stack. One exceptional case with power electronics is considered for comparison, where an MPPT is used with a 95% efficiency and a cost of 0.3 $/W_DC_. Gas conditioning, separators, piping, pumps, and control systems are lumped in the electrolyzer balance of system (BOS) cost. Compressors, pumps, and heat exchangers costs are described by eqs S8–S16. Cost values are taken from various references^7,17,28,37,65^ and summarized in Table 4. Conversion from the $/W_p_ to the $/m^2^ unit is performed by eqs S17 and S18. It should be noted that AEC and PEMEC stack costs are changing rapidly.

**Table 4:** PV-EC System Costs

parameters	unit	value	references
Photovoltaic System			
PV module	[$/W_p_]	0.3	(7)
PV BOS			
cabling	[$W_p_]	0.1	(7,37)
mounting	[$/W_p_]	0.1	(7,37)
operation and maintenance	[$/kW_op_]	9.28	(17)
Alkaline {PEM} Electrolyzer System			
initial stack cost	[$/W_d_]	0.347 {0.66}	(28,65)
stack cost in 2030		70% of the initial stack cost	(28,65)
replacement cost		15% of the stack cost	(16)
BOS cost	[$/W_d_]	0.24 {0.275}	(28,65)
operation and maintenance	[$/kW_op_]	17	(17)
Utilities			
compressor	[$]	*C*_comp_(*Ẇ*_comp_)	(66,67)
intercooler	[$]	*C*_hx_(A)	(66,67)
Consumables			
electricity	[$/kWh]	0.05	assumed
water	[$/kg H_2_]	0.014	(17)
Others			
land	[$/m^2^]	2	(68)
indirect		10% of the initial direct cost	assumed and^6,7^
contingency		10% of the initial direct cost	assumed and^6,7^

The land cost is chosen as *c*_land_ = 2 $/m^2^,^68^ above the average cost in Spain (9700 €/ha^69^) and costs used by Pinaud et al. (500 $/acre). The required land area is given by the total PV area needed to meet the target averaged daily hydrogen production and multiplied by a land-use factor *f* = 0.22, which is determined such that the shadowing between adjacent PV panels is minimized for a one-axis tracker system localized in a specific geographical location.^6^

Deionized water cost is often neglected due to its negligible contribution to the total cost of the systems.^14,16^ As liquid-based and water vapor PEC systems are evaluated here, and water costs are considered and taken from an electrolyzer system evaluated by Yates et al.^17^

The indirect cost *C*_indirect_, including engineering, procurement, and commissioning, and the contingency cost *C*_cont_ of the PV-EC system are both assumed as 10% of the initial direct cost *C*_direct_(*t*_*o*_). These costs are lower compared to that of a PEC device because the PV-EC system is a more mature technology^6,7^ and therefore less penalized by uncertainty than the PEC systems. The PV-EC investment and O&M costs are detailed in Section S6 of the SI. Other studies assume a more conservative value of 20%.^70^

#### 2.3.2 PEC System

The costs of the various PEC components are summarized in Table 5. The photoabsorber cost is 55 $/m^2^, derived from the price (0.5 $/W_p_) and efficiency (11%) of thin-film triple junction silicon solar cells, considered as a proxy for the photoabsorber cost for nonconcentrating applications. For an III–V solar cell (considered as a proxy of the photoabsorber in design 3 and concentrating applications), the cost is 21,000 $/m^2^, assuming an efficiency of 30% and a device price of 70 $/W_p_ (for an assumed 200 kW/year production).^71^ The costs of the catalysts are evaluated utilizing data from the mineral commodity summaries.^72^ Costs of catalysts made of a mixture of elements are calculated as , where *x*_*i*_, *M*_*i*_, and *C*_*i*_ are the molar concentration, molar mass, and cost of element *i*. The catalyst loading depends on the operating current density of the PEC device.^16^ For an operating current density range of 10–100 mA/cm^2^, 10 μg/cm^2^ Pt and 20 μg/cm^2^ IrO_*x*_ are required.^16^ Costs of the bipolar plate and the gas diffusion layer (GDL) are taken from an NREL report.^73^ The piping cost is computed considering the hierarchical piping network proposed by James et al.^14^ The PEC learning curve and replacement cost are assumed to be identical to the electrolyzers. Costs of feedwater pumps, compressors, and heat exchangers are calculated with respect to the operating pressure. The PEC system’s operation and maintenance cost is assumed to be 3.2% of the initial PEC system cost. This value is commonly used for electrolyzer systems. Other PEC device costs and hard BOS (mounting, condensers, water level controllers, hydrogen sensors, and miscellaneous equipment (not including pump)) costs are taken from various references^6,7,16^ and adapted to our case study (utility operations are specific to the plant’s operating pressure). The PEC investment and O&M costs are described in detail in Section S7 of the SI.

**Table 5:** PEC System Costs

PEC device	value	unit	references
Photoabsorber Material			
3-j Si	55	[$/m^2^]	(15)
III–V	21,000	[$/m^2^]	(71)
HER or COER Catalyst			
Pt	38.58	[$/g]	(72)
Ag	0.8	[$/g]	(72)
OER Catalyst			
IrO_*x*_	173.61	[$/g]	(72)
TCO	1 (designs 1,2)	[$/m^2^]	(7)
glass	10	[$/m^2^]	(7)
metal contact	0.5	[$/m^2^]	(7)
assembly	20	[$/m^2^]	(7)
Housing			
PEC housing	20	[$/m^2^]	(7)
bipolar plate and gas diffusion layer	225 (design 3)	[$/m^2^]	(73)
Membrane			
nafion	500	[$/m^2^]	(7,16)
AEM (PiperION)	180	[$/m^2^]	(25)
wiring	1.21 (designs 1,3)	[$/m^2^]	(7)
PEC cost in 2030	70% of the initial cell cost		(28,65)
replacement cost	15% of the PEC device cost		(16)
PEC BOP			
hard BOS (nonconcentrated PA)	22.48	[$/m_Conc_^2^]	(7)
hard BOS (concentrated PA)	100	[$/m_Conc_^2^]	(74)
piping (water, oxygen, hydrogen)	*C*_pipe_	[$]	(14)
pump	*C*_pump_(*Ẇ*_pump_)	[$]	(66,67)
PSA	*C*_PSA_ (total flow rate)	[$/m^2^]	(75)
Utilities			
compressor	*C*_comp_(*Ẇ*_comp_)	[$]	(66,67)
intercooler	*C*_*hx*_(A)	[$]	(66,67)
Consumables			
electricity	0.05	[$/kWh]	assumed
water	0.014	[$/kg H_2_]	(17)
CO_2_	0.04	[$/kg CO_2_]	DOE^76^
Others			
land	2	[$/m_conc_^2^]	(68)
indirect	10% of the initial direct cost		(6,7,70)
contingency	20% of the initial direct cost		(6)
operation and maintenance	3.2% of the initial direct cost		(16)
concentrator	100	[$/m_conc_^2^]	(77)

Table 5 shows that several components’ costs are high, and reducing their material use in the system is key to minimizing the hydrogen production price. It is the case for the membrane (in designs 1 and 2), the membrane-separated electrocatalyst assembly (in design 3), the bipolar plate (in design 3), and the gas diffusion layer (in design 3). The current concentration factor *F* is utilized to reduce a component’s area (membrane, electrocatalyst) with respect to the photoabsorber. The current density is then increased by *F* from the photoabsorber to the component. A typical current concentration factor of 10 is utilized to increase the EC cell current density. This allows that hydrogen production costs can be decreased.

The BOP of PEC design 3, which is operated with water vapor, is simplified. From Table 5, water level controllers, feedwater pumps, and water pipes are unnecessary. The hard BOS cost decreases by 13%.

For concentrating technologies with axis tracking, the concentrator cost and its maintenance is 100 $/m^2^^77^ and corresponds to that of a parabolic trough. This is different from the Fresnel lens concentrator technology used in concentrated PV (CPV). However, Fresnel lenses might not be applicable for PEC devices, given the larger areas considered.^19^ The concentrator concentrates light into the PA, which allows reducing the area of the PA by a factor *C* = *A*_C_*/A*_PA_. This is of interest for expensive and efficient PA, such as III–V materials. The BOS cost of the concentrator also corresponds to that of a parabolic trough^74^ and is normalized by the concentrator area.

For the reversible PEC operation, hydrogen storage is necessary within the boundary of the system as the produced hydrogen during photodriven electrolysis will be reused during the discharge phase. The storage is sized for 1000 kg H_2_ at 300 $/kg.

For the concentrated photodriven CO_2_ electrolyzer, the cathode (Ag) and anode (IrO_*x*_) loadings are 2 mg/cm^2^ and 1 mg/cm^2^, respectively,^26^ resulting in a total electrocatalyst cost of 1752 $/m^2^. The AEM cost is 180 $/m^2^. In this study, the cost of CO_2_ capture is contingent upon whether the CO_2_ is obtained from a dilute source (such as the environment) or a point source (such as chemical plants). The DOE has set a target cost of 40 $/t for CO_2_ capture, which is the value adopted for our analysis. At the outlet of the cathode, gaseous CO is present along with unreacted CO_2_ and hydrogen byproducts, which are also in gaseous form. To achieve efficient separation of CO from the other gases, the implementation of a pressure swing absorption (PSA) unit is necessary. To estimate the total flow rate at the cathode outlet to produce 1000 kg CO per day, a Faradaic efficiency of 95%, a single-pass conversion of 30%, and a carbonate formation of 30% are assumed (see eqs S35–S40). The PSA cost is .^75^ The CO is then compressed and stored at 50 bar and ambient temperature.

### 2.4 Optimization

A local search optimization algorithm is implemented to identify a good combination of decision variables that minimize the system LCOF (see Discussion S16). The relevant decision variables are defined for the (concentrated) PEC system and the PV-EC system, respectively
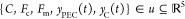
21a
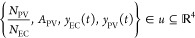
21b

For a system described by *X* variables, the optimization problem can be formulated as a minimization of LCOF(*X,u*) subject to *y*_*i*_(*t*) = [0,1], 0.01 ≤ *F*_c_ ≤ 10, 0.01 ≤ *F*_m_ ≤ 10, 1 ≤ *C* ≤ 1000, 0 ≤ *j*_EC,op_ ≤ 20,000 A/m^2^, . *y*_*i*_ is a replacement Boolean variable which, if activated, leads to a replacement of the concentrator, PEC, EC, and PV after half of the lifetime (i.e., 10 years). For PEC designs 1 and 2, *F*_c_ = 1 as catalysts are coated on the photoabsorber. For PEC design 3, *F*_c_ = *F*_m_ as catalysts are covered on the membrane. The latter constraint on the maximum current density in the EC can be replaced as
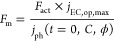
22where 0.1 ≤ *F*_act_ ≤1. For the CO_2_ electrolyzer, it is
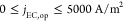
23

In the techno-economic analysis conducted by Schneidewind,^78^ a value of *C* ≤ 100 was employed. However, recent advancements have demonstrated large-scale hydrogen production from concentrated PEC devices above *C* = 800.^19^ Consequently, it is reasonable to assume that *C* ≤ 1000 for the purposes of the analysis. *C*, *F*_c_, and *F*_m_ are geometrical parameters that were already used by Rodriguez et al.^15^ and Dumortier et al.^8^ to optimize a multiobjective optimization problem, where the cost of hydrogen and the STH efficiency are simultaneously optimized. These variables were chosen as they allow for minimizing the area of expensive and efficient components over earth-abundant ones. Additionally, the set of binary variables *y*_PEC_(*t*), *y*_C_(*t*) indicates whether a component is replaced or not, taking into consideration degradation and cost of components.

#### 2.4.1 Uncertainty Assessment via the Monte Carlo Model

A Monte Carlo sampling method is implemented to address uncertainties related to the practical implementation of PEC devices. At each simulation, draws are realized from *X* random variables normally distributed (see Table 6) and sampled with the Monte Carlo method. For each draw, the local search optimization algorithm finds *u* that minimizes the LCOF. Both technical and economic parameters are used as inputs to the Monte Carlo simulation.

**Table 6:** Nominal and Standard Deviation of Normally Distributed Input Variables

parameter	nominal	standard deviation	unit	references
Technical Parameters				
GHI	630	15.75	[W/m^2^]	(79)
short current density at 1000 W/m^2^	71	2.8	[A/m^2^]	assumed
PA degradation (PEC design 3)				
open-circuit voltage	0.75	0.075	[%/year]	(52)
short current density	0.25	0.025	[%/year]	(52)
other degradation rates taken from^51^	Table S1	10% from nominal value	[%]	(51)
log of the anodic exchange current density	–6	1	[A/m^2^]	(45)
Economic Parameters				
PA cost	38.5	5.5	[$/m^2^]	(37)
membrane cost	500	50		(65)
catalysts cost	38.56	3.856	[$/m^2^]	(65)
Other PEC Components				
PEC components except GDL and b.p.	deterministic	10% of the nominal value	[$/m^2^]	(65)
B.p.+ GDL	225 (design 3)	22.5 (design 3)	[$/m^2^]	(65)
PEC cost scaling factor	0.9	0.03	Per 10-fold size increase	(17)
log of the system size	3.5	0.5	kg/day	assumed
PEC cost after 10 years	70	5	[%] of today’s PEC cost	assumed
water cost	4	1	[$/kL]	(17)
water usage	9	0.5	[L/kg H_2_]	(17)
PEC replacement cost	15	2	[%] of PEC’s cost at the time of replacement	assumed

As long-term GHI data are not available online for Seville, the standard deviation of the GHI is evaluated from a normal probability density function in Phoenix, Arizona, from 1961 to 2008.^79^ Phoenix was chosen due to its latitude proximity with Seville (37.3891°N for Seville and 33.4484°N for Phoenix). A standard deviation of 2.5% was identified. The capital cost scaling factor per 10-fold size increase varies from 0.9 with a standard deviation of 0.03.^17^ The log of the averaged daily solar fuel production is utilized to evaluate the influence of the system size on the LCOF. The daily averaged nominal fuel production is 1000 kg/day, corresponding to a ∼1 MW scale electrolyzer. The PA efficiency variation is modeled by varying its short current density. The thin-film efficiency of a typical module is 10%, with a 12.5% record efficiency reported by NREL. A standard deviation of 0.5% from the nominal efficiency (assumed as ∼11%) is assumed. PEC design 3 photoabsorber degradation mean value is 1%/year. Degradation values from 0.5 to 1.5%/year fall within a 95% confidence interval.^52^ Anodic exchange current densities reported in the literature vary by several orders of magnitude.^45^ The standard deviation is chosen to span through the range of reported values.

Learning curves for thin-film solar modules show that costs have decreased from 0.5 $/W_p_ in 2017 to 0.2 $/W_p_ in 2020. A nominal PA cost of 0.35 $/W_p_ is assumed with a standard deviation of 0.05 $/W_p_ (with costs converted in [$/m^2^] taking an 11% efficiency PV thin-film module). PEM costs range from 700 to 1300 Euro/kW, with a mean value of 1000 Euro/kW.^65^ We assume that the EC component’s costs vary with a standard deviation σ of 10% from the nominal value (3σ corresponding to 30%). Future PEC costs, based on expected EC costs, are varied with a standard deviation of 5% from the nominal value. The upper value would correspond to a scenario in which future research investment in EC would be less than the one predicted by the learning curve. The lower value, on the other hand, would correspond to a higher level of R&D that could bring costs further down. Physical performances would also change in the future. However, it is assumed to be constant over the plant’s lifetime. Other costs include PEC housing, metal contact, wires, assembly, and glass.

The discount factor is assumed to be 6%, although some studies consider 12%.^7,16^ In this study, each system is evaluated with the same discount factor.

Obtaining the solution to the nonideal diode equation is computationally expensive due to its implicit form. The Monte Carlo simulation implies 4000 runs to be statistically accurate (Figures S8 and S9), and an optimization loop is implemented for each run. Considering there are 20 operating years, the diode equation is solved more than 4000 × 20 times. Using the nonideal diode equation in the Monte Carlo simulation would require extensive computational resources. Alternative methods exist to solve the nonideal diode equation with limited computational resources. Here, an approximate single-diode model is (ASDM) used to explicitly express the current as a function of the voltage.^80^ The method and validation are shown in Figure S10.

**Figure 3:**
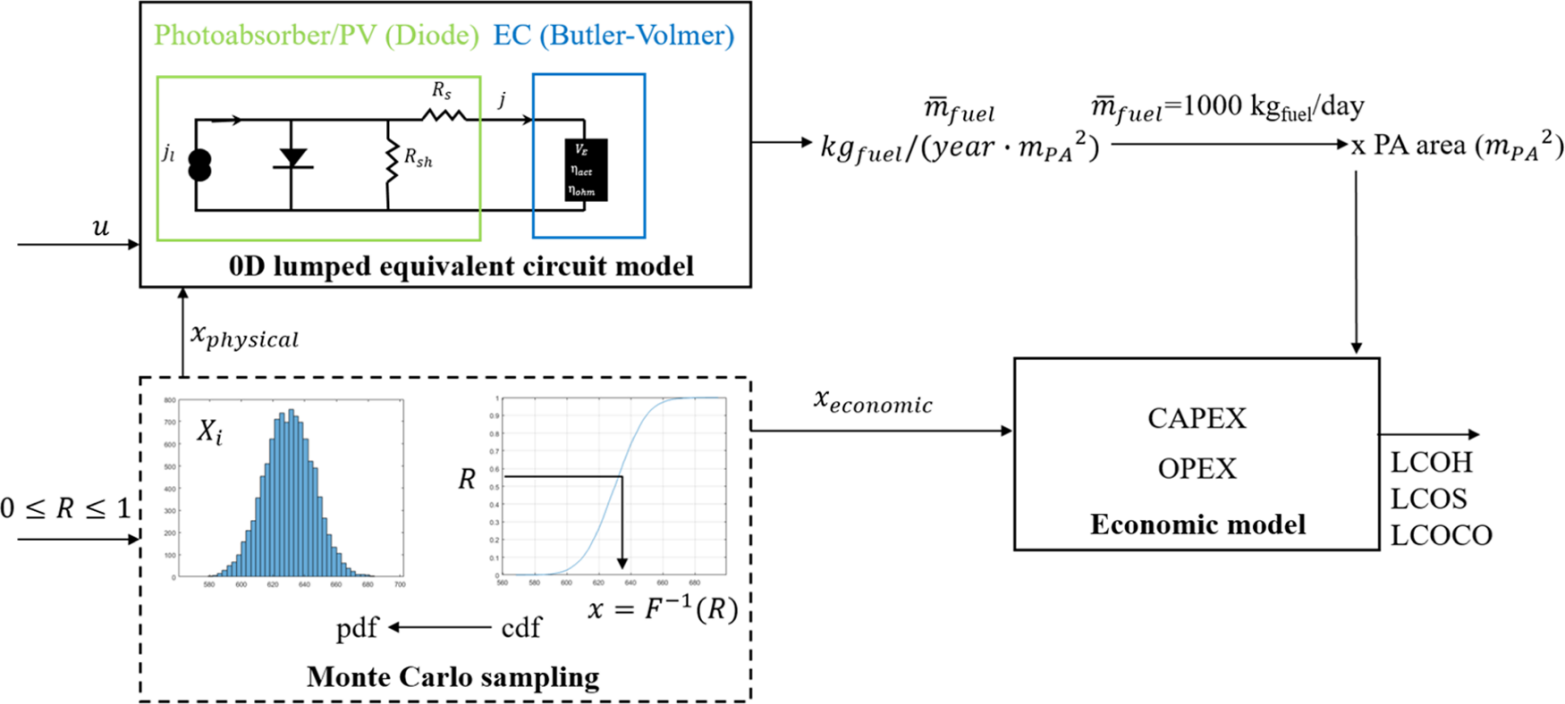
Flow diagram of the methodology.

Figure 3 summarizes the methodology of the work. The physical and decision variables are used as input to the performance model, which computes the yearly operating current density from the first to the 20th year. The hydrogen (or CO) fuel rate per unit of photoabsorber area is then computed using Faraday’s law. To meet the hydrogen or CO production requirement (i.e., 1000 kg H_2_/day or CO/day), the fuel rate per unit of photoabsorber area is multiplied by the required total photoabsorber area. The operational investment and total costs are computed in the economic model, where economic variables and the calculated total photoabsorber area are used as input to the model. The LCOH, LCOS, or LCOCO is then computed, taking into account the discount factor. In the probabilistic model, the physical and economic variables are sampled using the Monte Carlo sampling method. A random number *R* between 0 and 1 is first drawn. The sampled variable is then obtained by inversing the cumulative distribution function (cdf) of the variable’s probability density function (pdf), which is assumed to be normally distributed.

### 2.5 Future Costs

Future costs can be predicted using Wright’s law^81^ based on cumulative production capacity
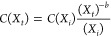
24where *b* is related to the learning rate LR = 1–2^–*b*^. *X*_*t*_ is the cumulative production at year *t* and *C*(*X*_*t*_) is the cost for a production volume of *X*_*t*_. Index *i* refers to the initial time. A learning rate of 18% is considered for PEM electrolyzers.^28^ This learning rate applies to core components of the PEM electrolyzer (membrane, bipolar plate, gas diffusion layers). Other EC components, which are relatively cheaper, have learning rates of 5 or 8%.^82^ A learning rate of 18% is considered for the whole EC stack for simplicity. The cumulative electrolyzer capacity was assumed to be *X*_*i*_ = 1 GW, corresponding to alkaline-based electrolyzers installed in 2020. The learning rate for c-Si modules is 32% from 2006 to 2020. A more conservative value of 25% is assumed, corresponding to the learning rate over the past 40 years. The learning rate for thin-film modules is 30% from 2006 to 2020.^37^ CdTe technology dominates the thin-film market, with a 6.1 GWp production in 2020. During the same period, the production of a-Si accounts only for 0.2 GWp and tends to become marginal. However, in this study, it is assumed that silicon-based thin-film technology regains interest in the framework of PEC development due to its higher open-circuit voltage. A learning rate of 18%, similar to PEMEC, is chosen for the PEC device and assumed identical for each of its components (the photoabsorber learning rate is selected with a more conservative value of 18% instead of 30%, the same as the EC components).^37^ The EC and PV O&M costs are assumed to decrease with a 10% learning rate.^83^ For PEC devices, the O&M is 3.2% of the initial direct cost and decreases over time as the PEC device cost decreases with a learning rate of 18%. The PEC devices’ future costs are calculated based on the future cumulative electrolyzer capacity. Different scenarios evaluating future electrolyzer manufacturing capacities can be found in the IRENA^84^ report and study by Schmidt et al.^28^ From these references, the following growth scenarios are considered: (i) 0.36 GW/year and (ii) 2.5 GW/year capacity growth and a target of (iii) 1 TW and (iv) 5 TW cumulative installed capacity in 2050, encompassing the International Energy Agency projection of 3.6 TW.^85^ For PV module growth, the base scenario from Vartiainen et al.^83^ is selected, where the cumulative capacity in 2050 is ∼20 TW. The PV and EC (and PEC) cumulative capacities of these scenarios are shown in Figures S11 and S12. In this study, future system costs are predicted until 2040. The year corresponds to the system installation, i.e., a plant installed in 2040 will be operating until 2060, with possible component replacement in 2050. The balance of plant, utility costs, and technical parameters are assumed to be constant over time.

## 3 Results and Discussion

### 3.1 Current Deterministic Cost for Water-Splitting Application

**Figure 4:**
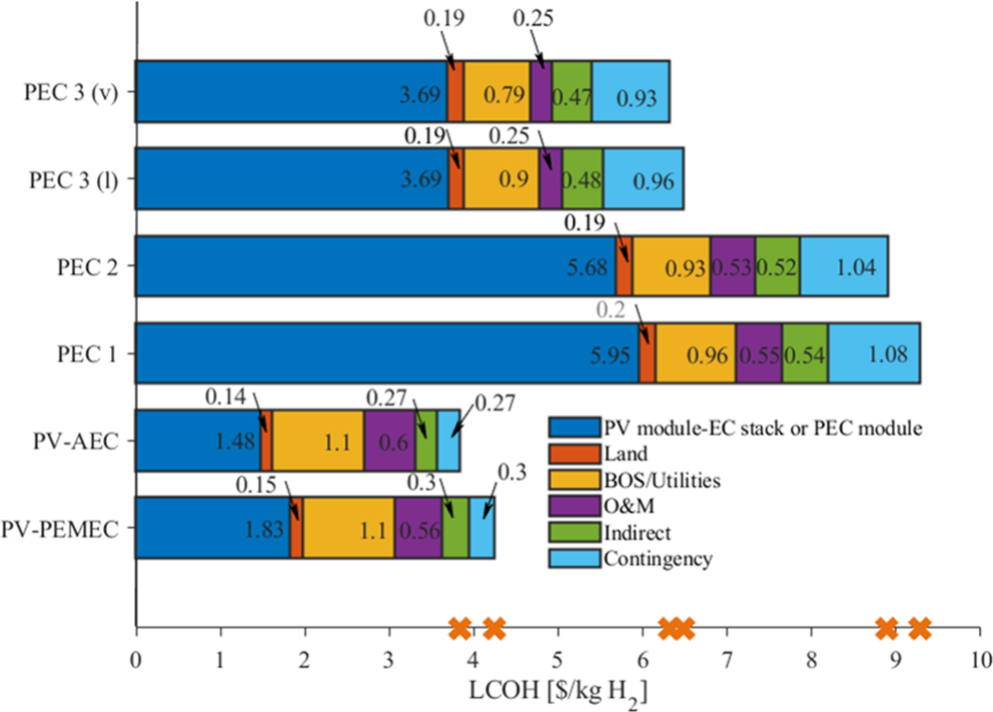
Deterministic LCOH cost of the PEC system with the three different designs, design 3 operated with liquid and vapor feed and PV-EC systems with AEM or PEM membrane design. Orange crosses indicate total LCOH values for each system, and the bar shows individual contributions of module, land, BOS, utility, O&M, indirect, and contingency costs. The LCOHs are 3.86, 4.24, 6.32, 6.47, 8.89, and 9.28 $/kg_H_2__ for PV-AEC, PV–PEMEC, PEC 3 (v), PEC 3 (l), PEC 2, and PEC 1, respectively.

The optimized LCOH for two PV-EC systems (PV–PEMEC, PV-AEC) and three PEC designs (including the water vapor operation of PEC design 3) are shown in Figure 4. For PV-EC systems, the LCOH is minimized for *y*_EC_ = 0 (no EC replacement). For PEC designs 1 and 2, the LCOH is minimized for *y*_PEC_ = 1 and *F*_m_ = 10, i.e., the PEC device is replaced after 10 years of operation due to a large decrease in performance (the STH efficiency decreases from 8 to 5% and to 6.6% in 10 years for PEC designs 1 and 2, respectively). For PEC design 3, *y*_PEC_ = 0 and *F*_m_ = 10. PEC design 3 degradation rates are less critical (the STH efficiency decreases from 8 to 7.7% in 10 years) than for designs 1 and 2, but is more expensive in the design (use of gas diffusion layers and bipolar plates), making replacement less advisable. PV-AEC emerges as the most economical system with 3.86 $/kg_H_2__, followed by PV–PEMEC with 4.24 $/kg_H_2__ due to higher degradation and more expensive components. Incorporating an MPPT increases the cost to 5.29 $/kg_H_2__. In the current PV-EC coupling configuration, the MPPT adds little improvement to the system as the operating point is chosen to be as close as possible to the maximum power point of the PV (see Figure S26). If the MMPT cost were to be 0 $/W_DC_, the cost would only decrease to 4.16 $/kg_H_2__ (i.e., less than a 2% decrease compared to a directly coupled PV-EC system). The area of the PV for the PV–PEM system is 308,720 m^2^ and for the PV-AEC system is 298,880 m^2^.

The PV areas for the PEC design 1, design 2, design 3(l), and design 3(v) systems are 431,600, 410,860, 396,630, and 397,030 m^2^, respectively.

PEC design 3 (v) stands out as the most competitive PEC design despite being 64% more expensive than the PV-AEC system. PEC designs 1, 2, and 3 (l) are 140, 130, and 68% more expensive than the PV-AEC system, respectively. The PV module-AEC stack cost only accounts for 1.48 $/kg_H_2__ (38% of total cost), whereas the PEC module cost of design 3 (v) is 3.69 $/kg_H_2__ (58% of total cost), the most expensive part of the solar hydrogen plant. Indeed, while PV modules and EC stacks are separately optimized by choosing the number of EC cells and PV cells in series, such as to operate at the maximum power point (see Figures S1 and S2), PEC modules can only be optimized by varying the photoabsorber area with respect to that of the catalyst and are constrained to a practical design factor of *F ≤* 10. The high cost for Nafion and catalysts compared to that of the photoabsorber necessitates an optimized *F* value of 10. Even though EC overpotentials increase due to higher operating current densities, catalyst material reduction decreases the hydrogen production costs overall.

PEC design 3 is cheaper than other PEC designs for several reasons: (i) reduced degradation as the PA is not in contact with the electrolyte, (ii) no PEC replacement, and (iii) the catalysts are coated on the Nafion, which allows simultaneous reduction of the two expensive PEC components’ areas with respect to that of the PA (*F*_m_ = *F*_c_), whereas PEC designs 1 and 2 have catalysts coated on the PA (*F*_c_ = 1). The breakdown catalyst costs are shown in Figure S13, depicting large differences between the designs: 1.69, 1.23, and 0.08 $/kg_H_2__ for designs 1, 2, and 3, respectively (18, 14, and 1% of the total cost of the designs, respectively). PEC design 3 (v) is simpler in design (no pumps, fewer pipes, no water feedstock) than design 3 (l), which explains why the BOS cost is 10% cheaper for water vapor operation. Water vapor operation is not limited by its higher ohmic overpotentials (18% higher) associated with reduced membrane hydration. This is because the PA and EC *IV* curves intersect within (or close to) the PA plateau region. After 20 years of operation, the operating current densities are 35.77 and 35.72 A/m^2^ for PEC designs 3 (l) and 3 (v), respectively (see Figures S5 and S6). PV-EC systems are not cost competitive (LCOH > 20 $/kg_H_2__) when operated with water vapor as they are designed, in terms of catalyst loading, to operate at current densities above 1000 A/m^2^. PEC design 3 (v) competitiveness depends on its operating limiting current density, which depends on the ambient RH. The LCOH increases by 2% if the limiting current density decreases from 450 to 400 A/m^2^. Then, the cost increases by 30% and even up to 90% when the limiting current density decreases further to 300 and 200 A/m^2^, respectively (Figure S14).

Compared to others, we chose a discount factor of 6% and a yearly average GHI of 630 W/m^2^. Grimm et al.^7^ used a GHI of 800 W/m^2^ and a discount factor of 12%. Using these values would result in an LCOH of 5.69 $/kg_H_2__ for PEC design 3 (v), which is 10% less than the result in Figure 4. Keeping the same GHI of 630 W/m^2^ but increasing the discount rate to 8 and 12% would result in a LCOH of 7.35 and 9.57 $/kg_H_2_,_ respectively. Due to uncertainties associated with PEC systems, a discount rate above 6% would likely be warranted. If the economy of scale is considered with a scaling factor of 0.9 per 10-fold size increase, the design 3 (v) LCOH decreases to 4.95 $/kg_H_2__ when the plant’s size is scaled up and increased to 50 t_H2_/day. The cost of water can vary depending on the system’s location. While Yates et al. consider the water cost for an electrolyzer system to be 0.014 $/kg_H_2__, Glenk^86^ report 0.08 $/kg_H_2__, therefore increasing the water contribution cost to the LCOH from 0.2 to 1.2% for PEC 3(l). In water-scarce regions, the advantage of PEC 3 (v) becomes more pronounced over PEC 3 (l) as water cost increases. Additionally, it is worth noting that a pressure increase to 20 bar of the PEC device only marginally reduces the LCOH (less than 5%).

PV-AEC emerges as the most economical system due to the use of more affordable components. However, PEC 3 (v) shows promise as a competitive alternative with its BOS costs (1.1 and 0.79 $/kg_H_2__ for PV-AEC and PEC 3(v), respectively, corresponding to 28% and 13% of total cost, respectively) and O&M costs (0.6 and 0.25 $/kg_H_2__ for PV-AEC and PEC 3(v), respectively, corresponding to 16 and 4% of total cost, respectively) being lower than that of the PV-EC system. Material cost reduction can potentially provide an advantage to PEC devices for future solar hydrogen production.

### 3.2 Current Probabilistic Cost for Water-Splitting Application

**Figure 5:**
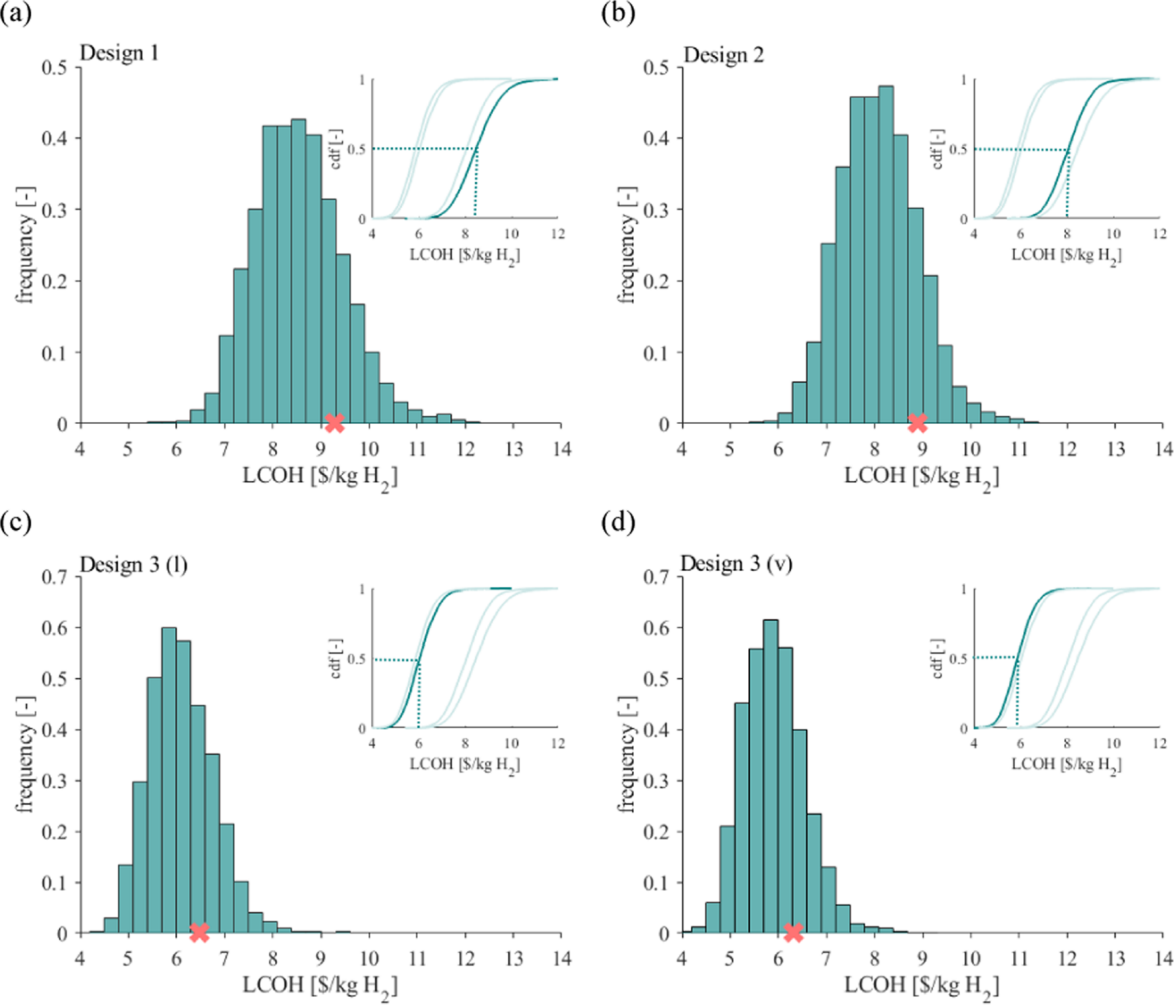
LCOH distribution and cumulative distribution function of the Monte Carlo simulation for PEC designs 1 (a), 2(b), 3 (l) (c), and 3 (v) (d). Orange crosses indicate deterministic LCOH.

The probabilistic LCOH for PEC designs 1, 2, 3 (l), and 3 (v) are shown in Figure 5. Their mean LCOH values are 8.53, 8.09, 6.1, and 5.91 $/kg_H_2__, respectively, with standard deviations of 0.92, 0.81, 0.7, and 0.64 $/kg_H_2__. The mean yearly averaged STH efficiency is 6.7, 6.9, 7, and 7%, which is lower than the usual 10% efficiency assumed for PEC devices in the literature due to degradation being considered and more conservative thin-film module efficiencies assumed in this study. PEC 3 (v) achieves the lowest minimum LCOH of 3.95 $/kg_H_2__, although it remains higher than the deterministic PV-AEC cost. We observed that PEC design 3 is more promising than other designs in reaching cost competitiveness with PV-EC systems. The cdfs of the four designs show that PEC designs 1 and 2 have less than a 10% probability of producing hydrogen at an LCOH of less than 7 $/kg_H_2_._ In contrast, designs 3 (l) and 3(v) have more than 90% probability of producing hydrogen at an LCOH of less than 7 $/kg_H_2__. PEC designs 1 and 2 have a 50% probability of producing hydrogen at an LCOH lower than 8.5 and 8 $/kg_H_2__, respectively, while for PEC 3 (l) and 3 (v), the LCOH drops down to 6 and 5.9 $/kg_H_2__, respectively.

**Figure 6:**
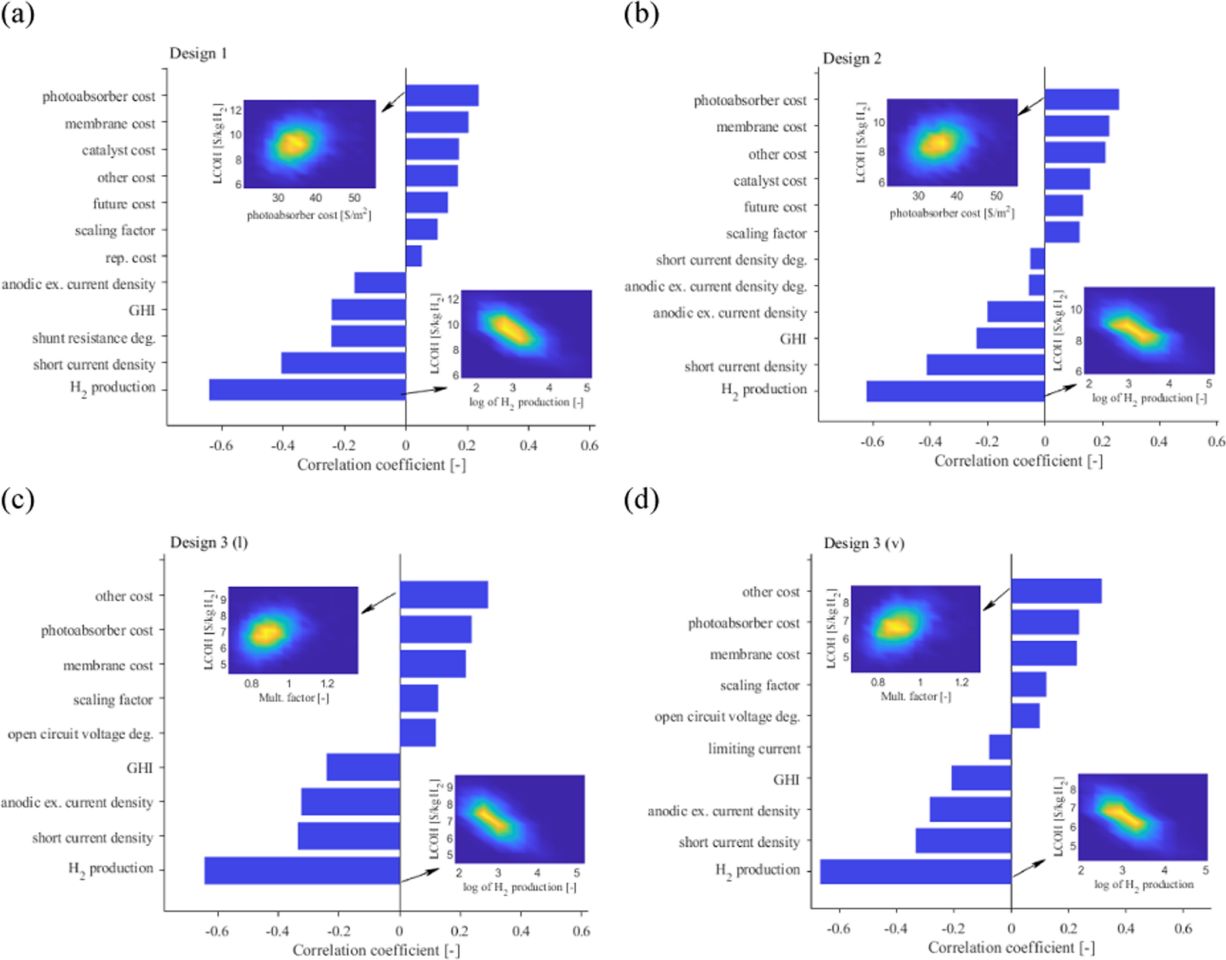
Correlation of the input variables with the LCOH for PEC designs 1 (a), 2 (b), 3 (l) (c), and 3 (v) (d).

Figure 6 shows the correlation of the different input variables with the LCOH. The two contour plots in each subfigure show the density of data points for LCOH with respect to the input variable for the largest positive and negative correlation coefficients. The correlation coefficient can be derived from a regression slope observed in these data points. The scale of the hydrogen plant (H_2_ production rate) shows the highest correlation (∼−60 to −65%) with the LCOH for all PEC designs. Increasing the plant size will lead to an LCOH reduction due to the economy of scale. The variable showing the second highest correlation with the LCOH is the PA’s short current density correlation (∼−35 to −40%), equivalent to an increase in PA’s efficiency. Increasing the short current density increases the operating current density and consequently the hydrogen production rate. The increase is linear if the PA and EC *IV* curves intersect in the PA *IV* plateau region. The third input variable that more importantly and negatively influences the LCOH is the electrical contact degradation for design 1, the GHI for design 2, and the anodic exchange current density for PEC designs 3 (l) and 3 (v). This is because design 1 is subjected to strong wire and metallic contact degradation, whereas design 2 is exposed to less light than other designs because the membrane is also irradiated. Designs 3 (l) and 3 (v) entail no replacements during their operational lifetime. However, the PA and EC *IV* curves intersect in the PA declining *IV* curve region (see Figure S5) toward the end of the plant’s lifetime. To remedy this decreasing operating current density, a catalyst with a higher exchange current can decrease the activation overpotentials and can potentially shift the operating point to a higher current density. The PA and other PEC costs show the highest positive correlation with the LCOH (∼25% for PEC designs 1 and 2 and ∼30% for PEC designs 3 (l) and 3 (v)). For PEC designs 1 and 2, the PA is the most expensive part of the PEC device when *F* = 10. For PEC designs 3 (l) and 3 (v), the most expensive PEC part corresponds to other PEC costs, in which the bipolar plate and GDL are lumped.

In short, the probabilistic analysis reveals that PEC design 3 (v) exhibits the lowest LCOH, demonstrating its potential competitiveness with the PV-EC system, with cost reduction primarily influenced by the hydrogen production scale and PEC component cost.

### 3.3 Future Deterministic PEC Cost for Water-Splitting Application

While the current hydrogen cost of PEC-derived hydrogen remains high, there is potential for future cost reduction. This reduction can be achieved by leveraging learning curve predictions associated with the increased cumulative capacity installation of PVs and ECs, as predicted in various scenarios (see eq 24). Anticipatory scenarios for future PEC system installation capacity are currently not available. Therefore, scenarios pertaining to ECs are employed as a substitute method to estimate the prospective costs of PEC devices.

**Figure 7:**
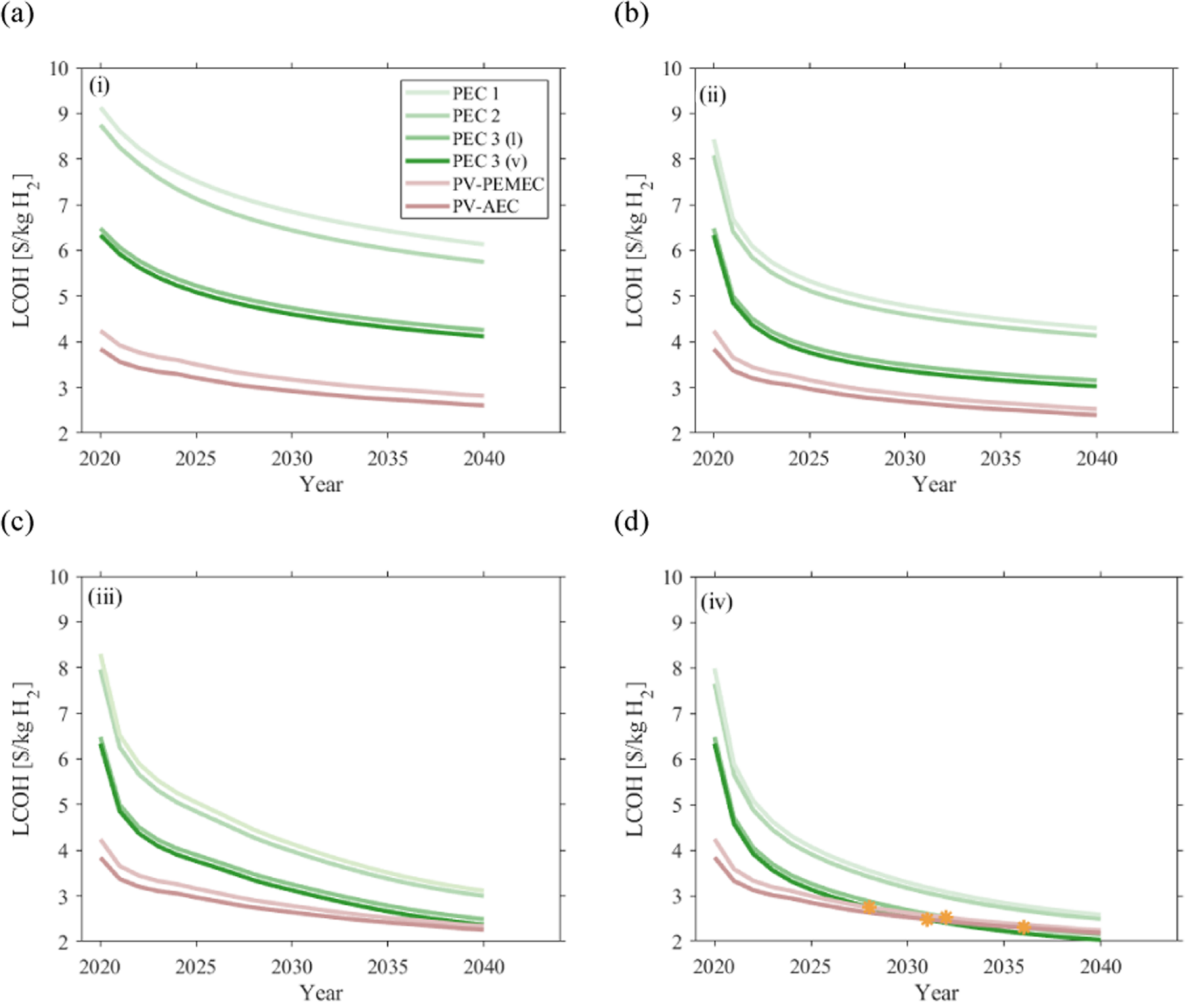
LCOH prediction from 2020 to 2040 for PEC designs 1, 2, 3 (l), 3 (v), PV–PEMEC, and PV-AEC considering four different expert capacity growth scenarios, designated by i, ii, iii, and iv in the manuscript, respectively: (a) 0.36 GW/year and (b) 2.5 GW/year capacity growth, and a target of (c) 1 TW and (d) 5 TW cumulative installed capacity in 2050. The stars in (d) indicate when the PEC systems become more competitive than the two PV-EC systems.

Figure 7 shows the LCOH projection from 2020 to 2040 for four expert scenarios evaluating future PEC deployment. In scenario (i), in which the annual electrolyzer deployment rate is 0.36 GW_el_ (i.e., a cumulative capacity of 8.2 GW_el_ by 2040), PEC devices neither achieve cost competitiveness with PV-EC technology nor achieve the target of 2$/kg_H_2__. In 2040, the LCOH is 4.12 and 2.6 $/kg_H_2__ for PEC design 3 (v) and PV-AEC, respectively. Future predicted learning rates lie between 29 and 43% for the c-Si PV module.^87^ If a future learning rate of 35% is considered, instead of the historically based 25% learning rate, the PV-AEC LCOH becomes 2.45 $/kg_H_2__ in 2040 (Figure S25a). In scenario (ii), the annual electrolyzer deployment rate is 2.5 GW_el_ (i.e., a cumulative capacity of 51 GW_el_ by 2040). An increase of 250% of the cumulative installed capacity is observed from (if this scenario was implemented in the year 2020) year 2020 (with 1 GW_el_ cumulative installed capacity) to the year 2021 (i.e., a 3.5 GW_el_ cumulative installed capacity). This explains the higher slope of the LCOH curve at the beginning, which decreases as the installed cumulative capacity only increases by 71% from 2021 to 2022, then by 42% from 2022 to 2023, etc. In this scenario, the LCOH of PEC designs 3 (v) and PV-AEC are 3 and 2.4 $/kg_H_2__, respectively. It can also be observed that the LCOH in 2020 for PEC designs 1 and 2, which are being replaced after 10 years, is ∼7% less expensive than in scenario (i). This is because PEC devices being replaced after 10 years are cheaper in scenario (ii). In the third scenario, with 1 TW of (P)EC installed cumulative capacity by 2050, the LCOH of PEC designs 3 (v), PV–PEMEC, and PV-AEC is 2.37, 2.34, and 2.26 $/kg_H_2__, respectively. PEC design 3 cost competitiveness is almost achieved. In the last scenario, with 5 TW of EC installed cumulative capacity by 2050, PEC design 3 (v) achieves cost competitiveness with PV–PEMEC in 2028 and PV-AEC in 2031. PEC design 3 (l) reaches cost competitiveness with PV–PEMEC in 2032 and PV-AEC in 2036. PEC designs 1 and 2 remain more expensive in 2040 than the PV-EC technology. In 2040, PEC design 3 (v) also achieves the target of producing hydrogen for 2 $/kg_H_2__ as the only technology (PV-EC does not achieve it unless a learning rate of 35% is utilized for the c-Si PV module in the PV-AEC system (see Figure S25b)) or device type (device 1, 2, and 3 (l) do not achieve it).

**Figure 8:**
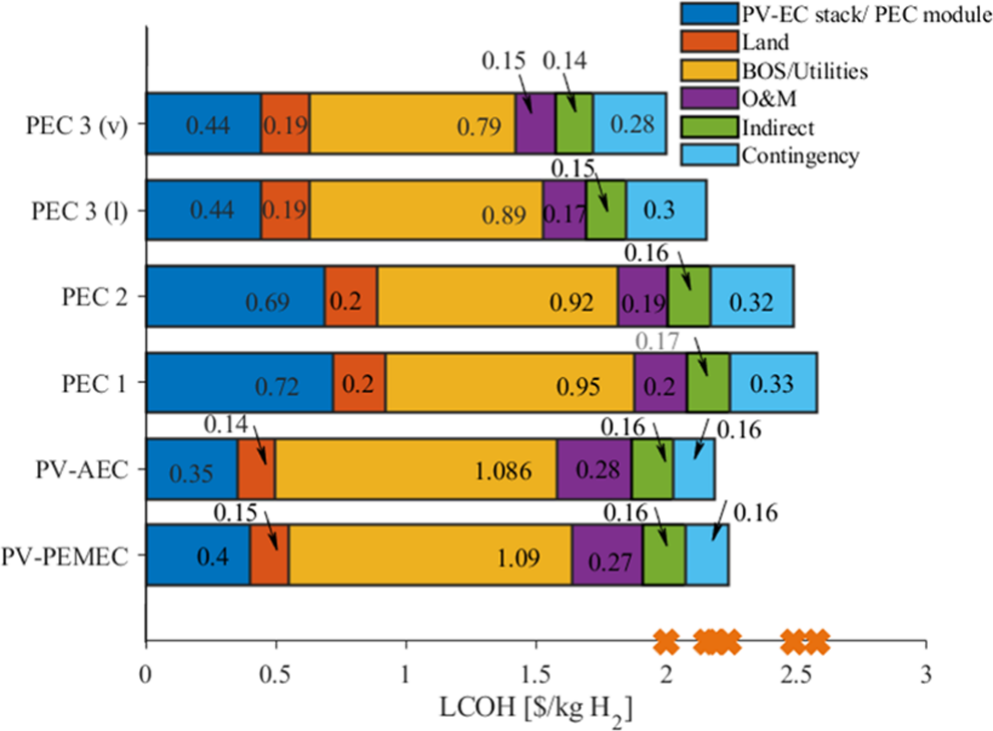
Stacked deterministic LCOH cost of PEC and PV-EC systems in 2040 considering scenario (iv). Orange crosses indicate total LCOH values for each system. Orange crosses indicate the LCOH for the various approaches: 1.99, 2.14, 2.18, 2.23, 2.47, and 2.57 $/kg_H_2__ for PEC 3 (v), PEC 3 (l), PV-AEC, PV–PEMEC, PEC 2, and PEC 1, respectively.

The PEC design 3 becomes competitive with PV-EC technology once the PEC material cost is sufficiently small and plant utilities dominate the LCOH. Figure 8 shows the PEC and PV-EC cost breakdowns in 2040 for scenario (iv). PEC designs 3 (l) and 3 (v) are cheaper than PV–PEMEC and PV-AEC. Even though PEC design 3 material cost is 0.44 $/kg_H_2__ (22% of the total cost for design 3 (v) and 21% for design 3 (l)), which is 11 and 26% more expensive than the PV–PEMEC and PV-AEC stack, respectively, BOS, utilities, and the O&M costs are cheaper for PEC systems. BOS/utility costs are 1.086, 1.09, 0.89, and 0.79 $/kg_H_2__ for PV-AEC, PV–PEMEC, PEC 3 (l), and PEC 3 (v), respectively (corresponding to 50, 49, 40, and 42%, respectively, of total cost). BOS/utilities costs are more important in this scenario than PEC material and PV-EC stack costs. Simpler PEC system’s utilities/BOS make this technology suitable for a scenario where a large capacity of systems would be installed. The O&M costs are also cheaper for PEC designs (0.15–0.2 $/kg_H_2__ corresponding to 7.5–7.9% of total cost) than for PV-EC (0.27–0.28 $/kg_H_2__ corresponding to 12.1–12.8% of total cost) as PEC requires maintenance of only one technology while PV-EC requires it for both the PV module and the EC stack. Less piping installed and no pump utilization also leads to a ∼ 0.1 $/kg_H_2__ reduction in utilities/BOS for PEC 3 (v) compared to PEC 3 (l).

Currently, the PEC-based water-splitting solutions are predicted not to be competitive with PV-EC systems. Water vapor-fed PEC systems hold the potential for industrial-scale applications, provided that mass transport challenges are addressed. This necessitates the development of novel PEC designs; among them, a design based on porous photoelectrodes has shown promising results.^62^ Additionally, it is important to explore alternative chemicals or applications that can leverage this technology, such as utilizing it for CO_2_ reduction or operating the CIPEC device reversibly (i.e., producing hydrogen and reusing it for producing electrical power in the same reactor).

Table S4 summarizes the advantages and disadvantages of PEC and PV-EC systems outside the scope of techno-economics that could benefit additional (niche) applications (e.g., space missions).

### 3.4 Alternative Designs and Utilization: Concentrated Light, PEC for Storage, and CO_2_ Reduction

The following three alternative design variations are considered to provide pathways for cost-competitiveness of PEC systems: (i) PEC design 3 (l) with III–V PAs utilizing concentrated light for water splitting, (ii) reversible operation (i.e., photodriven electrolysis (EC) and fuel cell (FC) mode) of PEC design 3 (l) with III–V PAs and utilizing concentrated light, and (iii) PEC design 3 (l) with III–V PAs utilizing concentrated light and for CO_2_ reduction into CO. All applications are optimized for cost by varying *C*, *y*_pec*,*_*y*_C_, and *F*_act_.

For case (i), the LCOH is systematically minimized for *C* = 1000 across all combinations of (*y*_pec_, *y*_c_), as shown in Figure S16. Operating the PEC system under high irradiation concentration enables operation at a high current density (>1 A/cm^2^) despite achieving maximum degradation rates in the EC, as indicated in Table S1. Consequently, the system operates in the falling region of the PA *IV* curve. Substituting the PEC device allows the restoration of its initial performance, albeit at a higher replacement cost, as illustrated in Figure S17. Notably, in this specific study, the combination of (*y*_pec_, *y*_c_) = (1,0) enables current hydrogen production at an LCOH of 3.59 $/kg_H_2__, bringing the cost closer to the target of 2$/kg_H_2__ and being more cost competitive than PV-AEMEC systems.

Case (ii) holds significance not only for compact devices designed to achieve high power output while minimizing mass (as, for example, required in space missions) but also for energy storage purposes. It enables hydrogen production during high irradiation periods, which can then be reused within the same device to generate power. Employing concentrated light and III–V PAs in a reversible PEC device eliminates the need for charging costs, such as electricity purchases. The LCOS is minimized for a value of *C* = 1000 and the specific combination of (*y*_pec_, *y*_c_) = (0,0), as depicted in Figure S18, resulting in a storage cost of 0.2803 $/kWh. This cost optimization is achieved by utilizing a discharging/charging phase ratio of 2/3, as shown in Figure S19. These findings establish the concentrated reversible PEC device design as a cost-competitive option when compared to other storage technologies such as pumped-hydro, lithium-ion, flywheel, and vanadium redox-flow systems (0.15–0.8 $/kWh).^22^ Notably, it has the potential to be more cost-competitive than discrete electrolyzers and fuel cells and more competitive than dark URFC (0.308 $/kWh).^21^

**Figure 9:**
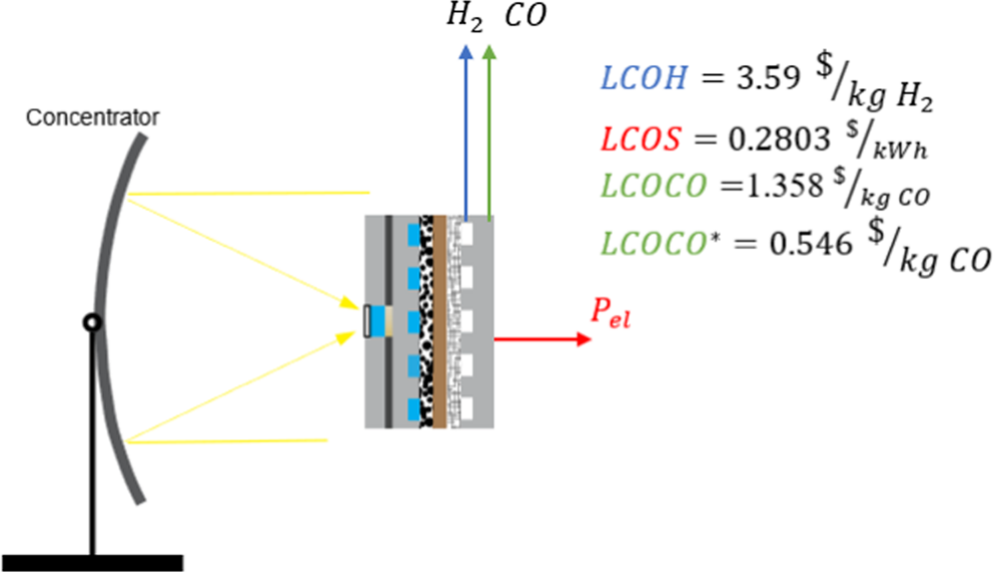
Deterministic LCOH, LCOS, and LCOCO cost of the PEC system utilizing concentrated radiation.

Case (iii) explores the possibility of utilizing an optical concentrator together with a PEC device to reduce CO_2_ into CO. The LCOCO is minimized for a value of *C* = 1000, as illustrated in Figure S20. However, due to high degradation rates, a replacement of the PEC device becomes necessary, as indicated in Figure S21. Without replacement, the total EC overpotentials would exceed the open-circuit voltage of the PA before reaching its end-of-life. The minimized LCOCO is determined to be 1.358 $/kg_CO_, which exceeds the market CO price of 0.6 $/kg_CO_. The primary contributors to this cost are the compression to 50 bar, the PSA process, and the CO_2_ purchase cost (see Figure S22). To reduce the cost, a sensitivity analysis is conducted, including sensitivity to the limiting current density, CO selectivity, single-pass conversion efficiency, CO_2_ cost, and compression ratio. When producing CO at 1 bar, the cost decreases to 0.797 $/kg_CO_. If the single-pass conversion efficiency drops from 30% (reference case) to 10%, the cost increases to 2.123 $/kg_CO_ (see Figure S23). Reducing the limiting current density from 500 to 100 mA/cm^2^ results in a noticeable increase in the LCOCO to 1.4853 $/kg_CO_. When increasing the limiting current density to 2000 mA/cm^2^, the effect on LCOCO is marginal. This is because the device is constrained by the maximum attainable optical concentration ratio, which consistently reaches *C* = 1000 (see Figure S20). These findings suggest that a current density of 500 mA/cm^2^ is the optimal target for CO_2_ electrolysis to achieve a favorable LCOCO. The sensitivity analysis enabled the identification of the most sensitive variables affecting LCOCO. By increasing the single-pass conversion efficiency to 50%, reducing the CO_2_ cost to 0.02 $/kg_CO_2__ (from 0.04 $/kg_CO_2__ at reference), and delivering the CO at 1 bar, the LCOCO* decreases to 0.546 $/kg_CO_. Figure 9 shows a schematic implementation of a concentrated PEC and summarizes the optimized cost for the three cases.

If the CIPEC device is located in Sevilla and not in Castello, the yearly average DNI increases from 630 to 715 W/m^2^. The LCOCO then decreases to 1.329 $/kg_CO_ (i.e., a 2% decrease) with (*y*_pec_, *y*_c_) = (1,0), the LCOS decreases to 0.258 $/kWh (i.e., an 8% decrease) with (*y*_pec_, *y*_c_) = (0,0), and the LCOH decreases to 3.25 $/kg_H_2__ (i.e., a 10% decrease) with (*y*_pec_, *y*_c_) = (1,0). The optical concentration is again *C =* 1000 for all cases.

In summary, including concentrated light with efficient but expensive III–V PA material allows to meet or exceed targeted cost metrics.

## 4 Conclusions

The economic competitiveness of solar hydrogen generation by photoelectrochemical (PEC) approaches was evaluated. Three realistic PEC device designs were evaluated and compared to two PV plus electrolysis approaches (PV–PEMEC and PV-AEC) at medium scales (production capacity of 1000 kg_H_2__/day).

The details of the PEC device design influence cost competitiveness and versatility, with the cheapest PEC device demonstrating an LCOH of 6.32 $/kg_H_2_,_ compared to 3.86 $/kg_H_2__ for the PV-AEC. Design 3 showed the most promise for cost reduction, with over 90% probability of producing hydrogen at an LCOH of less than 7 $/kg_H_2_._ Although initial material costs were comparatively higher, its reduced degradation profile poses advantages for prolonged operation in solar fuel plants compared to designs 1 and 2, which have less than a 10% probability of producing hydrogen at an LCOH below $7/kg H_2_. For design 3, vapour-fed operation showed to be more competitive than liquid water-fed operation, resulting from the lower BOS/utility costs.

Deploying a large-scale PEC plant is economically difficult at the present day. Even though PEC BOS/utilities and O&M costs are lower than the ones of a PV-EC system, PEC material costs are higher than PV modules coupled to EC stacks, which benefit from separate design optimization. An analysis considering various capacity growth scenarios suggests that a massive deployment (5 TW) of solar hydrogen devices by 2050 could position PEC systems as the most competitive technology by 2031, meeting the target of $2/kg_H_2__ in 2040. Notably, PEC design 3 (v) becomes cost-competitive once the PEC material cost is sufficiently small, so BOS/utilities become dominant in the LCOH. In 2040, the cost associated with PEC material accounts for 22% of the total cost, a significant decrease from the current 58%. With cost reduction, the performance and efficiency of individual components will likely also improve in the future (not considered here), further reducing the cost of hydrogen production. For instance, experimental PEC devices are already achieving STH efficiencies exceeding 10%, as demonstrated by Cheng et al., achieving 19% STH efficiency.^88^ Electrospinning techniques^89^ can contribute to fabricating nanofibrous structures of photoelectrode materials that enhance light absorption and charge transport, further reducing PEC material requirements.^62^ Such advancements in efficiency have the potential to significantly contribute to cost reduction. Moreover, it is worth mentioning that PEC systems possess a notable advantage: in the event of a malfunction in one cell, the remaining cells stay operational. In contrast, when a malfunction occurs in an EC stack, the entire system is rendered nonoperational until repairs are completed. This characteristic, particularly valuable for remote locations (especially when considering vapor-fed operation), could make PEC technology more appealing.

This study also explores three alternative present-day applications for PEC design 3 (l) devices, each employing an optical concentrator with III–V PA material: (i) concentrated PEC for hydrogen production, (ii) reversible concentrated PEC for fuel and power production, and (iii) concentrated PEC for CO_2_ reduction into CO. Using an optical concentrator allows to reduce the PEC material cost and enables a substantial reduction in the LCOH to 3.59 $/kg_H_2__. Furthermore, concentrated PEC devices can be employed in reversible operation, akin to dark unitized regenerative fuel cells, to facilitate energy storage, resulting in an LCOS of 0.2803 $/kWh. This competitive LCOS compares favorably with other established storage technologies, such as pumped-hydro (0.268 $/kWh), flywheel (0.683 $/kWh), lithium-ion (0.34 $/kWh), and vanadium redox-flow systems (0.289 $/kWh).^22^ Additionally, the concentrated PEC is predicted to produce CO with an LCOCO of 1.358 $/kg_CO_. Further improvements could potentially bring this cost down to 0.546 $/kg_CO_, achieved through increased conversion efficiency to 50%, selectivity,^90^ pressurized operations, and halving the CO_2_ capturing cost compared to the current target of 40 $/ton. This advancement results in costs below the market price of 0.6 $/kg_CO_. For instance, increased pressure operation or enhancing conversion efficiency through improved flow channel designs (such as porous flow-through electrodes^91^) offer room for further progress.

This versatility, combined with the potential to extend applications to more complex EC processes,^92^ positions concentrated PEC devices as a promising technology in renewable energy-powered electrochemical production. We acknowledge that techno-economic studies are sensitive to assumptions and that various unforeseen technological and policy advances can affect the results presented here. In any case, we do not encourage techno-economic studies, like the one presented here, to be used to justify (basic science) research priorities, but instead, they should help ignite innovation in technology development.

## Supporting Information Available

Additional details, equations, and parameter tables for the electrolyzer, degradation, Monte Carlo modeling, and the various cost models; detailed current–voltage results for the different systems; LCOH, LCOS, and LOOCO results for varying *C* and *F* factors; and detailed discussion on taxonomy and comparison of PV-EC vs PEC systems (PDF)
