# Supplementary material for: Probabilistic Techno-Economic Assessment of Medium-Scale Photoelectrochemical Fuel Generation Plants
Source: Energy Fuels. 2024 Jun 22;38(13):12058–77. doi: 10.1021/acs.energyfuels.4c00936 (PMC11228923; doi:10.1021/acs.energyfuels.4c00936)
Supplement: Supplementary file 1 — ef4c00936_si_001.pdf [file ef4c00936_si_001.pdf]

Probabilistic techno-economic assessment of medium-scale photo-electrochemical fuel generation plants

Alexandre Cattry, Hannah Johnson§, Despoina Chatzikiriakou§, Sophia Haussener*,[1]*

*École Polytechnique Fédérale de Lausanne (EPFL), Institute of mechanical Engineering, LRESE, 1015 Lausanne, Switzerland
§ Toyota Motor Europe NV/SA, Materials Engineering, Hoge Wei 33, 1930 Zaventem, Belgium

Supplementary material

S1. Electrolyzer performance model

The standard thermodynamic equilibrium potential is defined as [1]:

$$E_{0(\mathrm{l})}(T) = 1.23 - 0.9 \cdot 10^{-3}\,(T - 298) + 0.75\,R_{\mathrm{gas}}\,T\,ln(P)\,/F_{\mathrm{a}} \quad \text{(S1)}$$

where subscript l refers to liquid water. The thermodynamic equilibrium potential for water vapor splitting is [2]:

$$E_{0(\mathrm{g})}(T) = 1.253 - 2.452\,\cdot\,10^{-4}\,T + 0.75\,R_{\mathrm{gas}}\,T\ln(P)\,/F_{\mathrm{a}} \quad \text{(S2)}$$

To account for activation overpotentials, the Butler-Volmer expression is rearranged as [3]:

$$\eta_{\mathrm{act}} = \frac{R_{\mathrm{gas}}\,T}{2F_{\mathrm{a}}\alpha_{\mathrm{a}}}\,ln\left[1 + \frac{j}{j_{\mathrm{o,a}}}\right] + \frac{R_{\mathrm{gas}}\,T}{2F_{a}\alpha_{\mathrm{c}}}\,ln\left[1 + \frac{j}{j_{\mathrm{o,c}}}\right] \quad \text{(S3)}$$

The ohmic overpotential is defined as

$$\eta_{\mathrm{ohm}} = Rj \quad \text{(S4)}$$

The resistance of the PEMEC corresponds to the ionic resistance of a 127 µm thick Nafion membrane. At ambient temperature, the ionic conductivity of the Nafion membrane is 11 S/m. The ionic resistance of the alkaline electrolyzer is the combination of the 30% wt. KOH electrolyte and the Zirfon solid membrane [4]:

$$R_{30\%\mathrm{wt.KOH}} = \frac{d_{\mathrm{m}}}{\sigma_{\mathrm{KOH}}} \quad \text{(S5)}$$

$$R_{\mathrm{Zirfon}} = \frac{0.06 + 80e^{-T/50}}{10000} \quad \text{(S6)}$$

where d_{m}=2.5 mm and corresponds to the distance between two electrodes. The ionic conductivity, in unit of [S/m], is:

$$\sigma_{\mathrm{KOH}} = 100\left(-2.04m - 0.0028m^2 + 0.005332mT + \frac{207.2m}{T} + 0.001043\,m^3 - 0.0000003m^2T^2\right) \quad \text{(S7)}$$

where the molarity m is 6.9 mol/l. The thickness of the Zirfon material is assumed 0.5 mm.
The concentration overpotentials are calculated considering the limiting current density, assumed as 20% higher than the nominal operating current density described in Table 2.

S2. Current-voltage curves

The operating point of PV-EC (PEC devices) corresponds to the intersection between the PV module (photo-absorber) and the EC stack (membrane-separated electrocatalysts) current-voltage curves. In the following, operating points are shown by red dots for each year (20 years in total). The degradation of the current-voltage curves is qualitatively shown with a gradient of green (initial curve) to yellow (operation after 20 years) colors. For the PEC device, the current density is given per unit of the photo-absorber area. The current density of the membrane-separated electrocatalysts can be retrieved by multiplying the current density by the factor F_{m} or F_{c}.
The following figures, Figures S1-S6, show the current-voltage characteristics corresponding to the minimized deterministic LCOH cost shown in Figure 4.

[1] Corresponding author, e-mail: sophia.haussener@epfl.ch

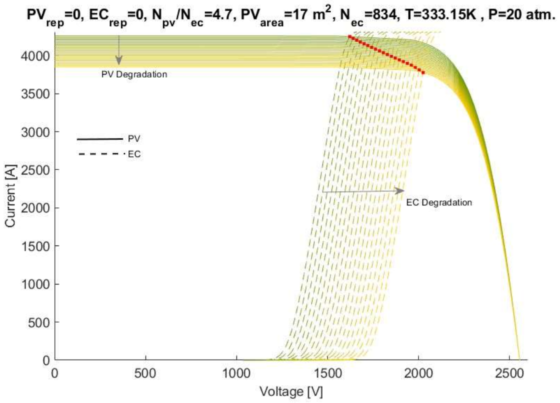

Figure S1. Yearly averaged PV-PEMEC current-voltage curves over 20 years. The red dots show the operating point for each year. The total PV area corresponds to the parallel arrangement of PV cells. The optimized number of PV modules and the number of EC cells are shown at the top of the figure. PV modules and EC modules are not replaced over the plant's lifetime. The total area ratio between PV and EC is 399.5.

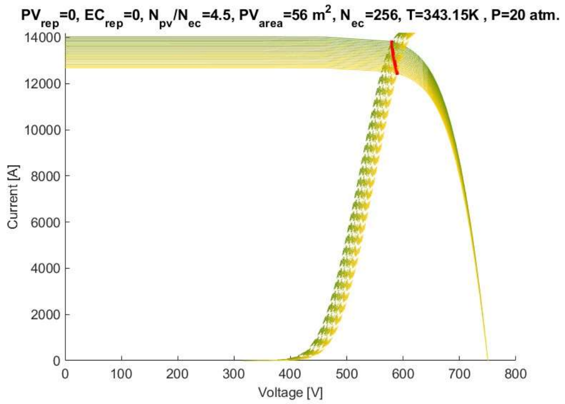

Figure S2. Yearly averaged PV-AEC current-voltage curves over 20 years. The total PV area gives the number of PV cells in parallel. The optimized number of PV modules and the number of EC cells are shown at the top of the figure. PV modules and EC modules are not replaced over the plant's lifetime. The total area ratio between PV and EC is 126.

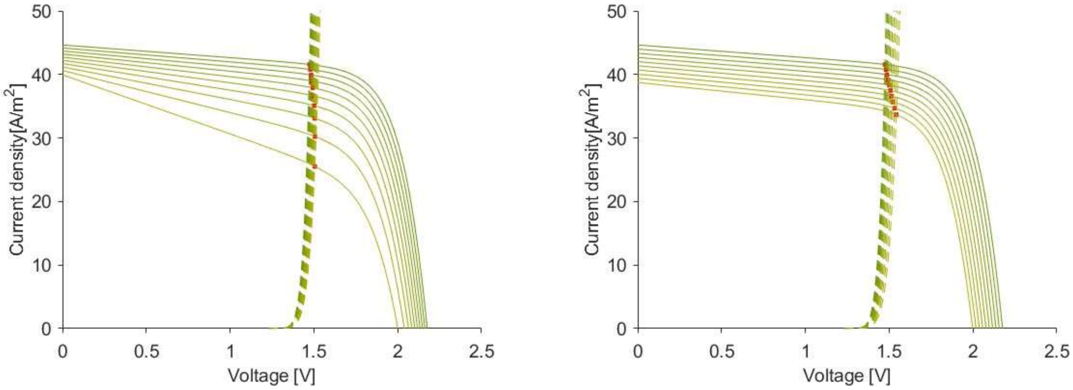

(a) b)

Figure S3. a) Yearly averaged PEC design 1 current density voltage curves over 20 years. Replacement of PEC components occurs after 10 years. The membrane area is 10 times smaller than that of the photo-absorber (F_m=10). b) Yearly averaged PEC design 2 current density-voltage curves over 20 years. Replacement of PEC components occurs after 10 years. The membrane area is 10 times smaller than the photo-absorber (F_m=10).

Figure S4 shows the yearly averaged PEC design 3 current density-voltage curves over 20 years. On the left, a PEC device was replaced after 10 years. On the right, the same PEC device operates over the plant's lifetime. It can be observed that beyond 30000 operating hours (i.e., 10 years), the photo-absorber behaves somewhat like a resistor. Although subject to higher degradation rates than crystalline silicon solar cells, thin film solar cells have a lifetime of more than 10 years. As the photo-absorber is not in contact with the electrolyte, it is assumed that the photo-absorber degradation behavior would be that of a thin film solar cell in the ambient environment. The electrical wire connecting the photo-absorber and the cathode is not a limiting parameter to PEC operation. It can be replaced at a negligible cost (wire cost (1.21 $/m^2$) <<PEC device cost) if the photo-absorber starts behaving like a resistor due to the shunt resistance increase. Figure S5 shows the yearly averaged PEC design 3 current density-voltage curves over 20 years, assuming thin film solar cell degradation rates (i.e., 1 %/year degradation rate). Figure S6 shows the yearly averaged PEC design 3 current density-voltage curves over 20 years with water vapor. A limiting current density of 450 A/m^2 on the membrane-separated electrocatalysts is assumed.

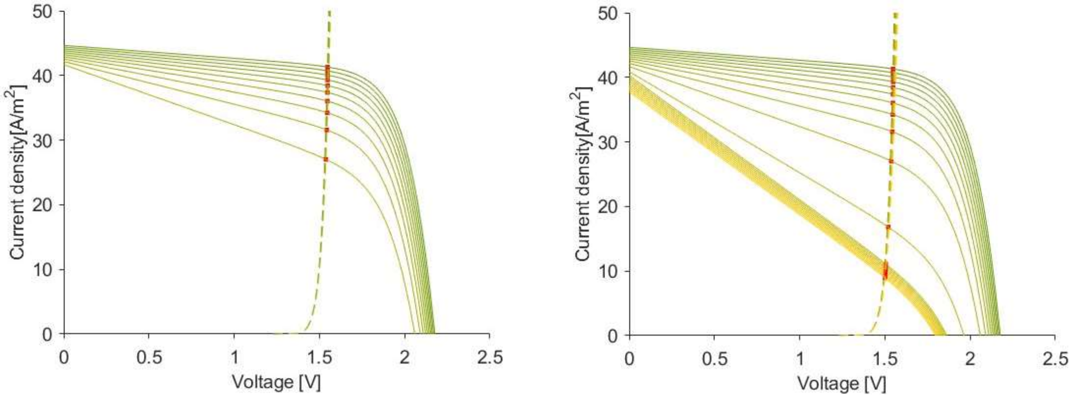

(a) (b)

Figure S4. Liquid water-based operation. a) Yearly averaged PEC design 3 current density-voltage curves over 20 years. Replacement of PEC components occurs after 10 years. b) Yearly averaged PEC design 3 current density-voltage curves over 20 years. PEC replacement is not considered over the plant's lifetime. The membrane-separated electrocatalysts area is 10 times smaller than the photo-absorber (F_m= F_c=10). PEC degradation rates are taken from [5].

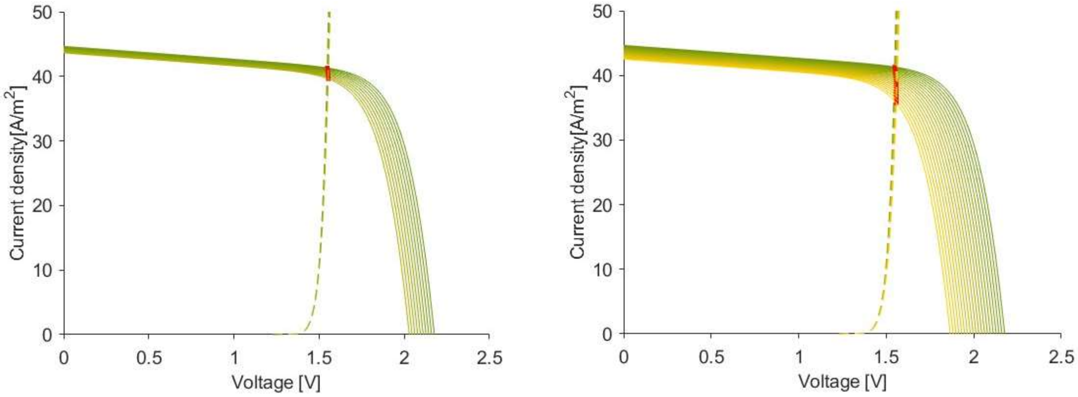

(a) (b)

Figure S5. Liquid water-based operation. a) Yearly averaged PEC design 3 current density-voltage curves over 20 years. Replacement of PEC components occurs after 10 years. b) Yearly averaged PEC design 3 current density-voltage curves over 20 years. PEC replacement is not considered over the plant's lifetime. The membrane-separated electrocatalysts area is 10 times smaller than the photo-absorber (F_m= F_c=10). Membrane-separated electrocatalysts' degradation rates are taken from [5], and PA degradation rates are taken from [6].

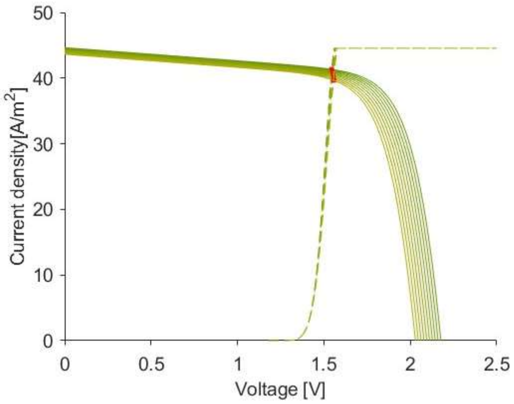

(a)

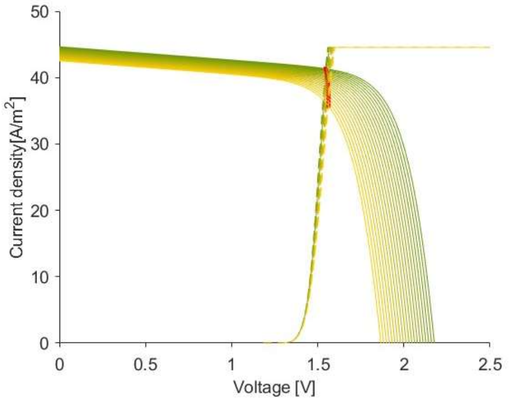

(b)

Figure S6. Water vapor-based operation. a) Yearly averaged PEC design 3 current density-voltage curves over 20 years. Replacement of PEC components occurs after 10 years. b) Yearly averaged PEC design 3 current density-voltage curves over 20 years. PEC replacement is not considered over the plant's lifetime. The membrane-separated electrocatalysts area is 10 times smaller than the photo-absorber (F_m= F_c=10). Membrane-separated electrocatalysts' degradation rates are taken from [5], and PA degradation rates are taken from [6].

S3. PEC degradation model

Table S1 shows the degradation rates of each parameter for three PEC designs taken from [5]. It is assumed that values are valid for $F_{m,c} \in [1,10]$. For $F_{m,c} \in]10,100]$, the degradation rates of the membrane and catalysts are increased and shown in red in the table. There is a discontinuity of degradation values at $F_{m,c}$=10. It is assumed that degradation rates increase per 10-fold $F_{m,c}$ increase. For $F_{m,c} \in [1,10]$, degradation is referred to as Min, and for $F_{m,c} \in]10,100]$ as Max. At $F_{m,c}$=10, Max. and Min. degradation values are used to calculate the LCOH. For PEC design 3 operating with water vapor, there is a 6.3% increase in LCOH when changing degradation from Min. to Max. For PEC design 3 operating with liquid water, there is a 4.07% increase in LCOH when changing degradation from Min. to Max. Table S2 shows the LCOH increase when each parameter varies from Min. to Max. The most significant increase is observed for the ohmic resistance of the PEC device operating with water vapor. Indeed, the ohmic resistance is more prominent for a PEC device operating with water vapor due to a lower ionic conductivity of the membrane.

Table S1. Degradation rates (x10^{-5} h^{-1}) for the three PEC designs [5]. Values for $F_{m,c} \in [1,10]$ and $F_{m,c} \in]10,100]$ are shown in black (Min) and red (Max), respectively. If there is a concentrator, the effective $F_{m,c}$ is obtained by multiplying by the concentrating ratio C.

Parameters	Design 1	Design 2	Design 3
Short current density	-0.35	-0.5	-0.2
Open circuit voltage	-0.2	-0.3	-0.1
Series resistance	3	0.5	3
Shunt resistance	-3	-1	-3
Anodic exchange current density	-1.35	-2.5	-0.25 (-1.35)
Cathodic exchange current density	-1.35	-2.5	-0.25 (-1.35)
Anodic tafel slope	0.5	0.5	0 (0.5)
Cathodic tafel slope	0.5	0.5	0 (0.5)
Ohmic resistance	4.4	4.4	4.4 (25)

Table S2. LCOH increases for varying each degradation parameter at a time from Min. to Max. (Table S1) for PEC design 3 operating with water vapor or liquid water at F_m=10.

Parameter	Liquid water operation	Water vapor operation
Anodic exchange current density	+0.31%	0.29%
Cathodic exchange current density	+0.19%	0.18%
Anodic tafel slope	+1.37%	1.42%
Cathodic tafel slope	+0.21%	0.2%
Ohmic resistance	+0.89%	2.7%

S4. Monte-Carlo ray tracing simulation

A Monte-Carlo ray tracing routine is implemented to compare light transmittance between PEC designs 1,3, and 2. For designs 1,3, three layers of materials with corresponding refractive indexes n are assumed: air (n=1) – glass (n=1.4835) – semiconductor (n=3.5). For design 2: air (n=1) – glass (n=1.483) – water (n=1.333) – TCO (n=1.89) – semiconductor (n=3.5). The radiation is assumed normally incident on the glass, i.e., the angle of incident radiation will not change when being reflected or refracted at different interfaces. It is assumed that the glass and TCO thicknesses are negligible, i.e., absorption is marginal. The water channel optical thickness ($\tau = \exp(-\alpha L) = \exp(-0.01\ \mathrm{cm}^{-1} 0.1\ \mathrm{cm} = 0.999)$ is negligible, and absorption can be neglected as well. Bubbles are assumed absent in the water channel. Anti-reflection coatings utilized to increase light transmittance (with ideal anti-reflection coating approaching $\sqrt{n_0 n_2}$) are not taken into account in the simulation. The reflectivity is calculated as: $\rho = \frac{(n_1 - n_{\mathrm{air}})^2}{(n_1 + n_{\mathrm{air}})^2}$

Algorithm S1. An iterative algorithm to determine the transmittance and reflectance

Initialize the total number of rays N_{rays};
Identify each material with a position: air pos=1, glass pos=2, etc.;
0. Initialize the position (air position pos=1) of the ray and its direction (vector direction downward dir=1);
1. Generate a random number R between 0 and 1;
If $R < \rho$
    If the ray is in the air
        The ray is reflected: $N_r = N_r + 1$;
        Go to step 0;
    Else
        Ray direction is $dir = -dir$;
    End
Else
    If ray in semiconductor
        The ray is transmitted: $N_t = N_t + 1$;
        Go to step 0;
    Else
        Ray is in $pos = pos + dir$;
    End
End

$$R = \frac{N_r}{N_{\mathrm{rays}}}$$

$$Tr = \frac{N_t}{N_{\mathrm{rays}}}$$

The Transmittance and reflectance for PEC designs 1, 3 and 2 are shown in Figure S7. The transmittances for 10^5 rays used in the simulation are 0.85 and 0.81 for PEC design 2 and PEC designs 1 and 3. Water acts as an anti-reflection coating as its refractive index is smaller than the one of the semiconductor but larger than the one of glass.

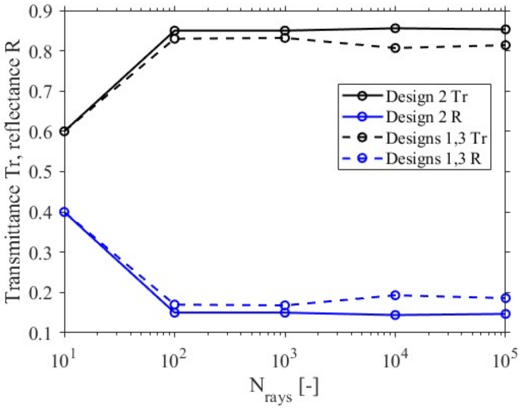

Figure S7. Transmittance and reflectance for PEC designs 1, 3 and PEC design 2 with respect to the number of rays used in the Monte-Carlo ray tracing simulation.

S5. Utilities cost model

The size of the compressors and heat exchangers are evaluated considering the first year of the plant's operation, i.e., when hydrogen production is the highest. The investment costs are computed with empirical correlations and sizing parameters found in the literature [7], [8].

Compressors. The high volumetric density of hydrogen at ambient conditions (12.1 m^3/kg) requires compressing the gaseous hydrogen to decrease the volume of storage needed. Multi-stage positive displacement compressors, such as piston-type compressors made in stainless steel 316L are selected for this study [9], [10]. The power consumption of an adiabatic compressor is estimated by:

$$\dot{W}_{\mathrm{comp}} = \frac{Z\,k}{\eta_{\mathrm{s,comp}}\,(k-1)}\,\dot{m}_{\mathrm{H_2}} R_{\mathrm{gas}}\;T_{\mathrm{in,comp}}\left(\pi_{\mathrm{comp}}{}^{(k-1)/k} - 1\right) \tag{S8}$$

where π_{comp} is the compression ratio, $T_{\mathrm{in,comp}}$ in Kelvin is the hydrogen temperature at the inlet of the compressor, $\eta_{\mathrm{s,comp}}$ = 0.85 is the isentropic efficiency, Z is the compressibility factor, R_{gas} is the universal gas constant, and k is the heat capacity ratio.

The energetic consumption of the motor utilized to drive the compression train is obtained by integrating the time-dependent power consumption over the operation time Δt and by considering a motor efficiency $\eta_{\mathrm{s,comp}}$ = 0.95:

$$W_{\mathrm{el,mot}} = \int_0^{\Delta t} \frac{\dot{W}_{\mathrm{comp}}(t)}{\eta_{\mathrm{mot}}}\,dt \tag{S9}$$

The operation cost over the operation time Δt of the motor utilized to drive the compressor corresponds to its electrical consumption. Assuming an electricity cost of c_{el}=0.05 \$/kWh, one obtains:

$$C_{\mathrm{el,comp}} = \int_0^{\Delta t} \frac{c_{\mathrm{el}}}{3600}\;\dot{W}_{\mathrm{el,mot}}(t)\,dt \tag{S10}$$

The installed investment cost of compressors is estimated with the Chauvel method [8]:

$$C_{\mathrm{comp}}(\dot{W}_{\mathrm{comp}}) = 16030\,\dot{W}_{\mathrm{comp}}{}^{0.4411}\,(f_{\mathrm{c,comp}}\,f_{\mathrm{g,comp}} + f_{\mathrm{mater,comp}} - 1) \tag{S11}$$

where $f_{\mathrm{c,comp}}$=1.2102 $\dot{W}_{comp}{}^{-0.091}$ is a general correction factor, $f_{\mathrm{g,comp}}$ = 2.72 is the mounting factor and $f_{\mathrm{mater,comp}}$= 1.7 is the material correction factor.

Heat exchangers. The high pressures in compressing hydrogen require using heat exchangers to sustain high-pressure differences between the shell and the tube sides. The spiral tube heat exchanger is therefore considered. The installed investment cost of spiral tube intercoolers is calculated based on the total heat exchange area and assuming counterflow heat exchange between the cold (water) and the hot (hydrogen) streams. The heat exchange area is estimated with the logarithmic mean temperature

difference (LMTD) method. An overall heat transfer coefficient of 300 W/m^{2}K is assumed. The investment cost of the heat exchangers is calculated from the total area of heat exchange [8]:

$$C_{\mathrm{HX}} = 4.6 \cdot 10^{3.4088+\ .6\ log_{10}(A)+0.09944\ (log_{10}(A))^2} \tag{S12}$$

Pumps. A stainless steel 316L pump is selected to compensate for pressure drop in pipes and pressurize the feed water. The pumping power is [8]:

$$\dot{W}_{\mathrm{pump}} = \frac{1.2\ \dot{m}_{\mathrm{feed}}(t)(\Delta P + P_{\mathrm{op}} - P_{\mathrm{atm}})}{\rho} \tag{S13}$$

where ρ is the water density, P_{op} is the operating pressure of the solar hydrogen reactor, and ΔP is the pressure drop in pipes (the pipe diameter can be adjusted to maintain a laminar flow). A 20% safety factor of over-capacity is utilized. The pressure drop in the pipes is calculated from the specific energy losses:

$$\Delta P = \rho g \Delta H \tag{S14}$$

where g is the gravitational constant on earth and ΔH is the head loss.

The operation cost over the operation time Δt of the motor utilized to drive the pump corresponds to its electrical consumption:

$$C_{\mathrm{el,pump}} = \int_0^{\Delta t} \frac{c_{el}}{3600}\ \frac{\dot{W}_{\mathrm{pump}}(t)}{\eta_{\mathrm{mot}}}\ dt \tag{S15}$$

Pumps' electrical consumption is negligible compared to other costs and can be neglected in this study.

The installed investment cost of compressors is estimated with the Chauvel method:

$$C_{\mathrm{pump}}(\dot{W}_{\mathrm{pump}}) = 808.19\ (\dot{W}_{\mathrm{comp}}/0.98)^{0.2724}\ (f_{\mathrm{c,pump}}\ f_{\mathrm{g,pump}} + f_{\mathrm{mater,pump}} - 1) \tag{S16}$$

where $f_{\mathrm{c,pump}}$=1.16 is a general correction factor, $f_{\mathrm{g,pump}}$ = 2.97 is the mounting factor and $f_{\mathrm{mater,pump}}$= 1.2 is the material correction factor.

Cost conversion

To convert from \$/W$_\mathrm{p}$ to \$/m^2 unit, the following operation is performed [11]:

$$c_{\mathrm{pv,module}}[\$/\mathrm{m}^2] = 1000[\mathrm{W/m}^2] \cdot \eta \cdot Cost\ PV\ [\$/\mathrm{W_p}] \tag{S17}$$

$$c_{\mathrm{pv,BOS}}[\$/\mathrm{m}^2] = 1000\left[\frac{\mathrm{W}}{\mathrm{m}^2}\right] \cdot \eta \cdot Cost\ BOS\ [\$/\mathrm{W_p}] \tag{S18}$$

where η is the efficiency of the photovoltaic module defined in technical parameters (Table 1). The PEMEC and AEC costs are taken with respect to the designed operating power.

S6. PV-EC investment and O&M costs

Investment cost. The initial investment cost is calculated at the initial time t_0. Components being replaced are discounted at the year of replacement. The total PV-EC system investment cost [\$] is defined as [2]:

$$C_{\mathrm{inv}} = C_{\mathrm{direct}} + C_{\mathrm{indirect}} + C_{\mathrm{cont}} \tag{S19}$$

where $C_{\mathrm{cont}} = 0.1 C_{\mathrm{direct}}(t_{\mathrm{o}})$, $C_{\mathrm{indirect}} = 0.1 C_{\mathrm{direct}}(t_{\mathrm{o}})$, and the direct cost is:

$$C_{\mathrm{direct}} = C_{\mathrm{PV,module}}(t_{\mathrm{o}}) + C_{\mathrm{PV,BOS}}(t_{\mathrm{o}}) + C_{\mathrm{land}}(t_{\mathrm{o}}) + C_{\mathrm{EC,stack}}(t_{\mathrm{o}}) + C_{\mathrm{EC,stack}}(t_{\mathrm{n/2}}) + C_{\mathrm{EC,BOS}}(t_{\mathrm{o}}) + C_{\mathrm{comp}}(t_{\mathrm{o}}) + C_{\mathrm{hx}}(t_{\mathrm{o}}) \tag{S20}$$

The PV module and PV BOS costs are defined as:

$$C_{\mathrm{PV,module}} = N_{\mathrm{PV,series}}(N_{\mathrm{PV,parallel}} A_{\mathrm{PV,module}}) c_{\mathrm{pv,module}} \tag{S21}$$

$$C_{\mathrm{PV,BOS}} = N_{\mathrm{PV,series}}(N_{\mathrm{PV,parallel}} A_{\mathrm{PV,module}}) c_{\mathrm{pv,BOS}} \tag{S22}$$

where $N_{\mathrm{PV,series}}$ is the number of PV modules in series, $N_{\mathrm{PV,parallel}}$ is the number of PV modules in parallel and $A_{\mathrm{PV,module}}$ is the area of a single PV module. The land cost is [12]:

$$C_{\mathrm{land}} = \frac{N_{\mathrm{PV,series}}(N_{\mathrm{PV,parallel}} A_{\mathrm{PV,module}}) c_{\mathrm{land}}}{f} \tag{S23}$$

The EC stack cost is:

$$C_{\mathrm{EC,stack}}(t_{\mathrm{o}}) = N_{\mathrm{stack}} j_{\mathrm{d}} V_{\mathrm{d}} c_{\mathrm{EC,stack}}(t_{\mathrm{o}}) \tag{S24}$$

$$C_{\mathrm{EC,stack}}(t_{\mathrm{n/2}}) = y_{\mathrm{EC}} N_{\mathrm{stack}} j_{\mathrm{d}} V_{\mathrm{d}} A_{\mathrm{EC,cell}} c_{\mathrm{EC,stack}}(t_{\mathrm{n/2}}) \tag{S25}$$

where N_{stack} is the number of cells in the stack, $j_{\mathrm{d}} V_{\mathrm{d}}$ is the EC nominal operating power, $c_{\mathrm{EC,stack}}(t_{\mathrm{o}})$ [\$/W$_\mathrm{d}$] is the initial stack cost, $c_{\mathrm{EC,stack}}(t_{\mathrm{n/2}})$ [\$/W$_\mathrm{d}$] is the stack cost at n/2 (i.e., at the time of

replacement), and y_{EC} is a binary decision variable (0,1) indicating wheatear the EC stack is replaced or not. The EC BOS cost is calculated as follows:

$$C_{\mathrm{EC,BOS}} = N_{\mathrm{stack}} j_{\mathrm{d}} V_{\mathrm{d}} c_{\mathrm{EC,BOS}} \quad \text{(S26)}$$

where $c_{\mathrm{EC,BOS}}$ [$/W$_{\mathrm{d}}$].

O&M cost. The O&M cost of the PV-EC system at year *t* is:

$$C_{\mathrm{O\&M}}(t) = C_{\mathrm{EC,O\&M}}(t) + C_{\mathrm{PV,O\&M}}(t) + C_{\mathrm{water}}(t) + C_{\mathrm{comp,el}}(t) \quad \text{(S27)}$$

where $C_{\mathrm{water}}(t) = c_{\mathrm{water}} m_{\mathrm{H2}}(t)$ is the water consumption. If $y_{\mathrm{EC}} = 1$, the O&M cost of the PV-EC system at year *t*=*n*/2 is:

$$C_{\mathrm{O\&M}}(t_{\mathrm{n/2}}) = C_{\mathrm{EC,O\&M}}(t_{\mathrm{n/2}}) + C_{\mathrm{PV,O\&M}}(t_{\mathrm{n/2}}) + C_{\mathrm{water}}(t_{\mathrm{n/2}}) + C_{\mathrm{comp,el}}(t_{\mathrm{n/2}}) + C_{\mathrm{EC,replace}}(t_{\mathrm{n/2}}) \quad \text{(S28)}$$

where $C_{\mathrm{EC,replace}}(t_{\mathrm{n/2}})$ is the EC stack replacement cost.

S7. PEC investment and O&M costs

Investment cost. The total PEC system investment cost [$] is defined as:

$$C_{\mathrm{inv}} = C_{\mathrm{direct}} + C_{\mathrm{indirect}} + C_{\mathrm{cont}} \quad \text{(S29)}$$

where $C_{\mathrm{cont}} = 0.2C_{\mathrm{direct}}(t_{\mathrm{o}})$, $C_{\mathrm{indirect}} = 0.1C_{\mathrm{direct}}(t_{\mathrm{o}})$, and the direct cost is:

$$C_{\mathrm{direct}} = C_{\mathrm{PEC,cell}}(t_{\mathrm{n/2}}) + C_{\mathrm{PEC,cell}}(t_{\mathrm{o}}) + C_{\mathrm{PEC,BOP}}(t_{\mathrm{o}}) + C_{\mathrm{pump}}(t_{\mathrm{o}}) + C_{\mathrm{pipes}}(t_{\mathrm{o}}) + C_{\mathrm{land}}(t_{\mathrm{o}}) + C_{\mathrm{comp}}(t_{\mathrm{o}}) + C_{\mathrm{hx}}(t_{\mathrm{o}}) \quad \text{(S30)}$$

where the hard BOS, pumps, and pipes costs are lumped in PEC BOP cost $C_{\mathrm{PEC,BOP}}(t_{\mathrm{o}}) = C_{\mathrm{PEC,Hard\,BOS}}(t_{\mathrm{o}}) + C_{\mathrm{pump}}(t_{\mathrm{o}}) + C_{\mathrm{pipes}}(t_{\mathrm{o}})$. The PEC cost is:

$$C_{\mathrm{PEC}}(t_{\mathrm{o}}) = A_{\mathrm{PA}} c_{\mathrm{PA}}(t_{\mathrm{o}}) + \frac{A_{\mathrm{PA}}}{F_{\mathrm{c}}} c_{\mathrm{HER}}(t_{\mathrm{o}}) + \frac{A_{\mathrm{PA}}}{F_{\mathrm{c}}} c_{\mathrm{OER}}(t_{\mathrm{o}}) + A_{\mathrm{PA}} c_{\mathrm{TCO}}(t_{\mathrm{o}}) + A_{\mathrm{PA}} c_{\mathrm{glass}}(t_{\mathrm{o}}) + + A_{\mathrm{PA}} c_{\mathrm{metal}}(t_{\mathrm{o}}) + A_{\mathrm{PA}} c_{\mathrm{assembly}}(t_{\mathrm{o}}) + A_{\mathrm{PA}} c_{\mathrm{housing}}(t_{\mathrm{o}}) + \frac{A_{\mathrm{PA}}}{F_{\mathrm{m}}} c_{\mathrm{BPL-GDL}}(t_{\mathrm{o}}) + \frac{A_{\mathrm{PA}}}{F_{\mathrm{m}}} c_{\mathrm{membrane}}(t_{\mathrm{o}}) + A_{\mathrm{PA}} c_{\mathrm{wiring}}(t_{\mathrm{o}}) \quad \text{(S31)}$$

If PEC replacement occurs at *t*=*n*/2 (i.e., $y_{\mathrm{PEC}} = 1$), $C_{\mathrm{PEC}}(t_{\mathrm{n/2}}) = 0.7C_{\mathrm{PEC}}(t_{\mathrm{o}})$.

O&M cost. The O&M cost of the PEC system at year t includes O&M costs of the PEC device, water consumption, and electrical consumption of compressors and pumps:

$$C_{\mathrm{O\&M}}(t) = C_{\mathrm{PEC,O\&M}}(t) + C_{\mathrm{water}}(t) + C_{\mathrm{comp,el}}(t) + C_{\mathrm{pump,el}}(t) \quad \text{(S32)}$$

where $C_{\mathrm{PEC,O\&M}}(t) = 0.032C_{\mathrm{direct}}(t_{\mathrm{o}})$. If $y_{\mathrm{PEC}} = 1$, the O&M cost of the PEC system at year *t*=*n*/2 is:

$$C_{\mathrm{O\&M}}(t_{\mathrm{n/2}}) = C_{\mathrm{PEC,O\&M}}(t_{\mathrm{n/2}}) + C_{\mathrm{water}}(t_{\mathrm{n/2}}) + C_{\mathrm{comp,el}}(t_{\mathrm{n/2}}) + C_{\mathrm{pump,el}}(t_{\mathrm{n/2}}) + C_{\mathrm{PEC,replace}}(t_{\mathrm{n/2}}) \quad \text{(S33)}$$

where $C_{\mathrm{PEC,replace}}(t_{\mathrm{n/2}})$ is the PEC stack replacement cost.

S8. Convergence test

The number of realizations required for the Monte-Carlo simulation is identified by performing a convergence test. The number of runs gradually increases until a smooth cumulative distribution function (cdf) is obtained. Figure S8 shows four Monte-carlo simulations of PEC design 1 using 250, 1000, 2000, and 4000 runs. The standard deviation of the LCOH with respect to the number of runs is shown in Figure S9. Both figures show that 4000 runs are sufficient to generate a smooth cdf curve.

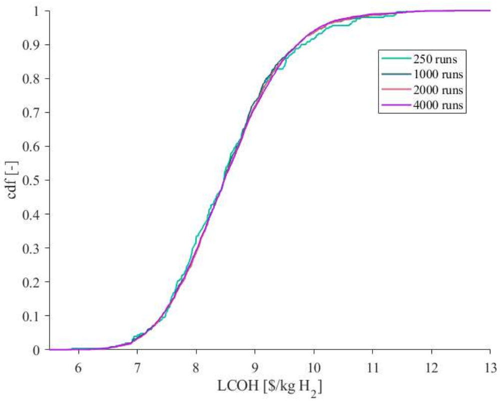

Figure S8. Four Monte-Carlo simulations of PEC design 1 using 250, 1000, 2000, and 4000 runs

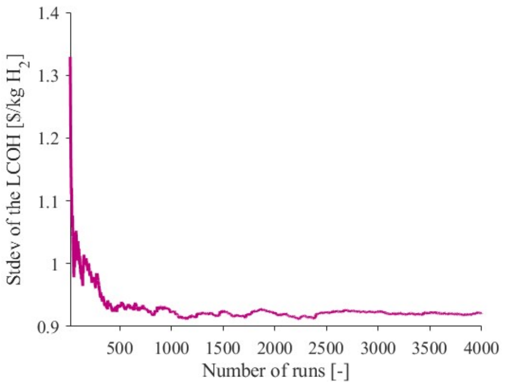

Figure S9. The standard deviation of the LCOH for PEC design 1 with respect to the number of runs performed in the Monte-Carlo simulation.

S9. Approximate Single-Diode model (ASDM)

In this section, the diode equation is approximated with the ASDM. The Current density is expressed as:

$$j = \frac{j_{\mathrm{sc}} - j_{\mathrm{o}} e^{\frac{V}{nV_t}} a_0 - V/R_{\mathrm{sh}}}{1 + j_{\mathrm{o}} e^{\frac{V}{nV_t}} a_1 + R_{\mathrm{s}}/R_{\mathrm{sh}}} \tag{S34}$$

More details about a_o, a_1 calculations can be found in [13]. $V_t = kT/q$.

The following Figure S10 compares the ASDM and the non-ideal diode model IV curves under standard test conditions (1000 W/m^2).

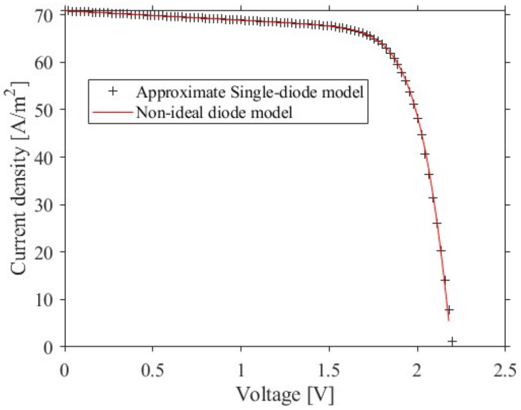

Figure S10. Validation of the ASDM model.

S10. PV and EC cumulative capacity

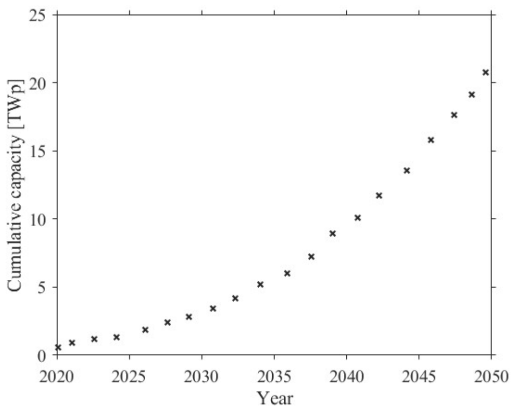

Figure S11. PV cumulative capacity from 2020 to 2050 (adapted from [14]).

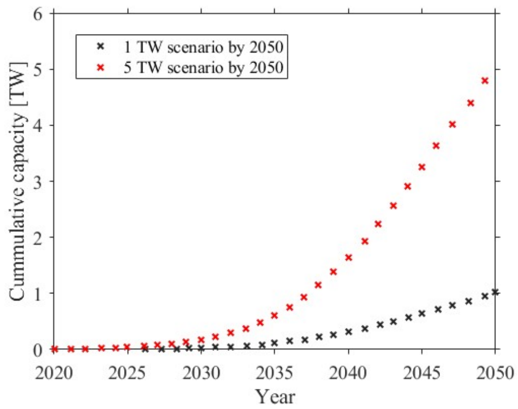

Figure S12. EC cumulative capacity from 2020 to 2050 is shown for a 1 TW and 5 TW cumulative capacity by 2050 (adapted from [15]). These same scenarios are assumed for the PEC growth scenarios.

S11. Current PEC cost breakdown

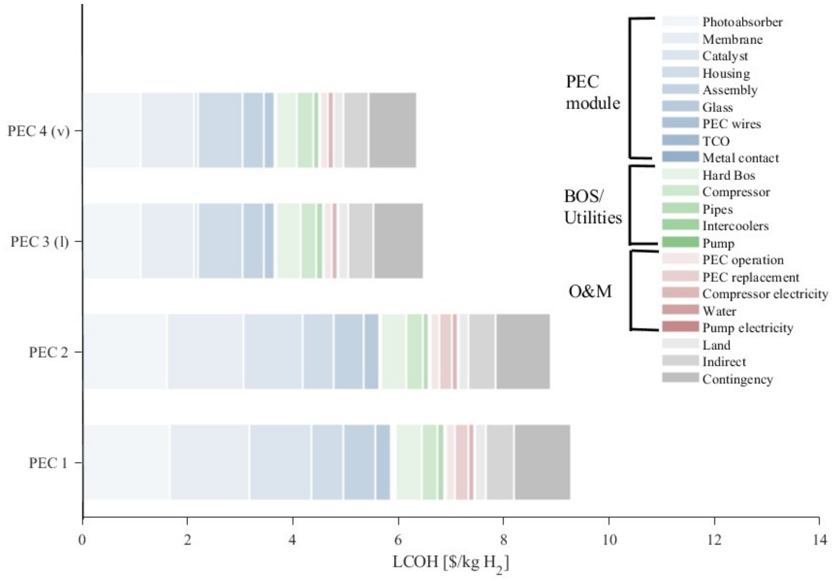

Figure S13. Current LCOH breakdown of the PEC module, BOS, and O&M for PEC devices.

S12. Influence of PEC design 3 (v) operating current density on the LCOH

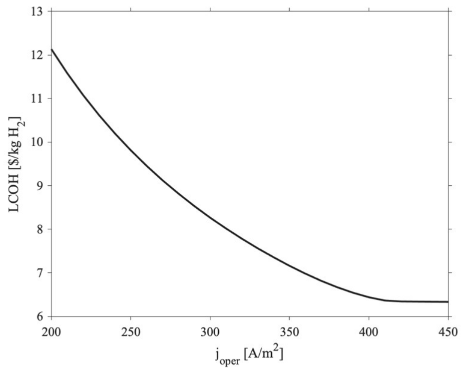

Figure S14. Deterministic LCOH of PEC design 3 (v) as a function of the designed operating current density (j_{limit}=1.1 j_{oper}).

S13. Occurrences of the binary optimization variables in the Monte-Carlo simulation

Figure S15 shows the number of occurrences of the PEC replacement decision variable in the Monte-Carlo simulation. For PEC design 1, y_{pec}=1 for 4000 runs. For PEC design 3(l), and (v), y_{pec}=0 for 4000 runs. For PEC design 2, y_{pec}=1 for 3637 runs out of 4000 runs. For the latter design, not replacing the PEC device after 10 years can be, in rare cases, cost-saving.

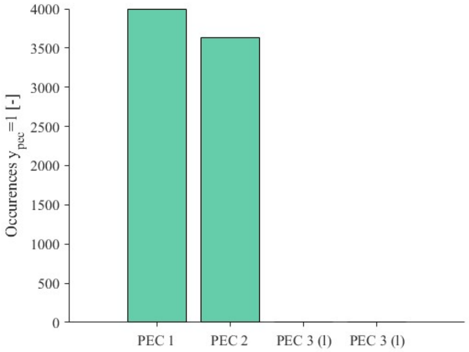

Figure S15. The number of PEC replacement decision variable occurrences in the Monte-Carlo simulation using 4000 runs.

S14. Alternative applications

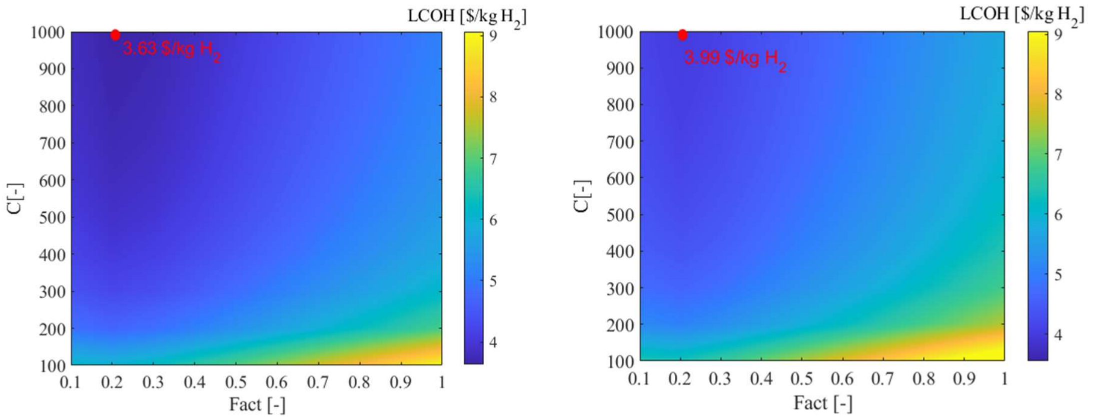

(a) (b)

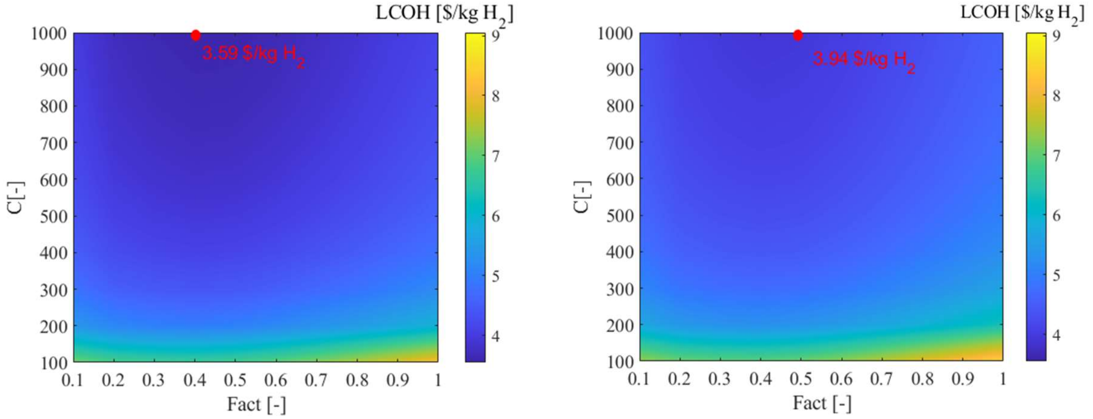

(c) (d)

Figure S16. LCOH contour plot as a function of irradiation concentration and current dilution from the PA into the EC for concentrated PEC water splitting utilizing PEC design 3 (l) considering $(y_{\mathrm{PEC}}, y_{\mathrm{C}})$ =(0,0) (a), (0,1) (b), (1,0) (c), (1,1) (d). The minimum cost is 3.63 $/kg H_2. Due to higher operating current densities at *C*=1000, increasing the catalyst loading might be required. Increasing the catalyst loading by 10 times results in a marginal cost increase of 3.73 $/ kg H_2.

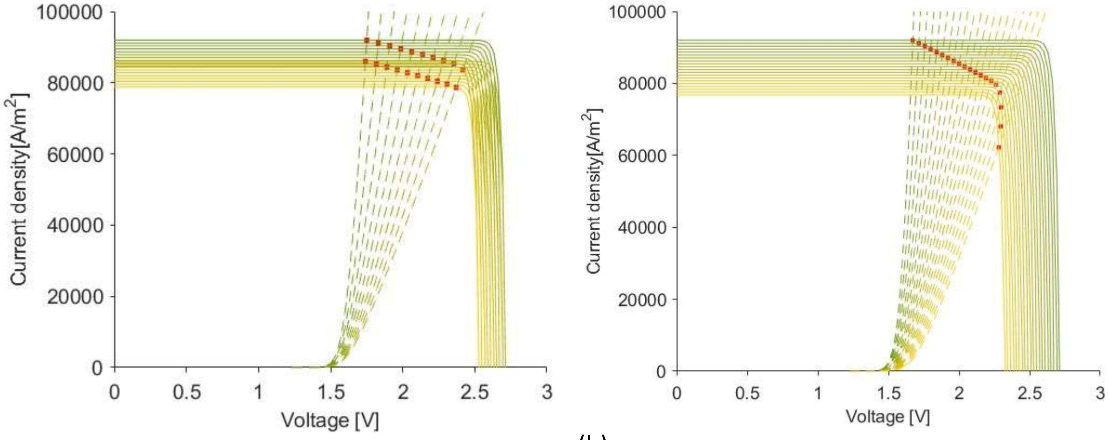

(a) (b)

Figure S17. Yearly averaged concentrated PEC design 3(l) current density voltage curves over 20 years. The red dots show the operating point for each year. The irradiation concentration is *C*=1000. a) $(y_{\mathrm{PEC}}, y_{\mathrm{C}})$ =(1,0), where replacement occurs after 10 years, and *Fact*=0.4. b) $(y_{\mathrm{PEC}}, y_{\mathrm{C}})$ =(0,0), and *Fact*=0.2.

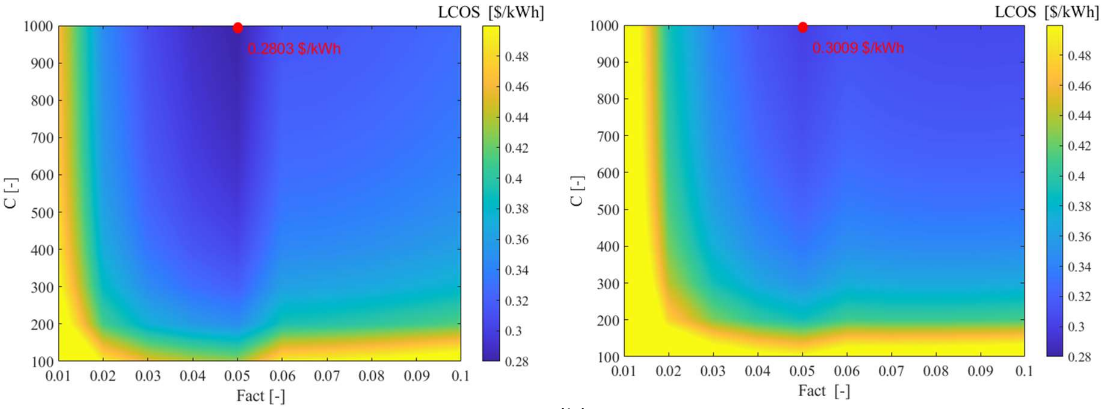

(a) (b)

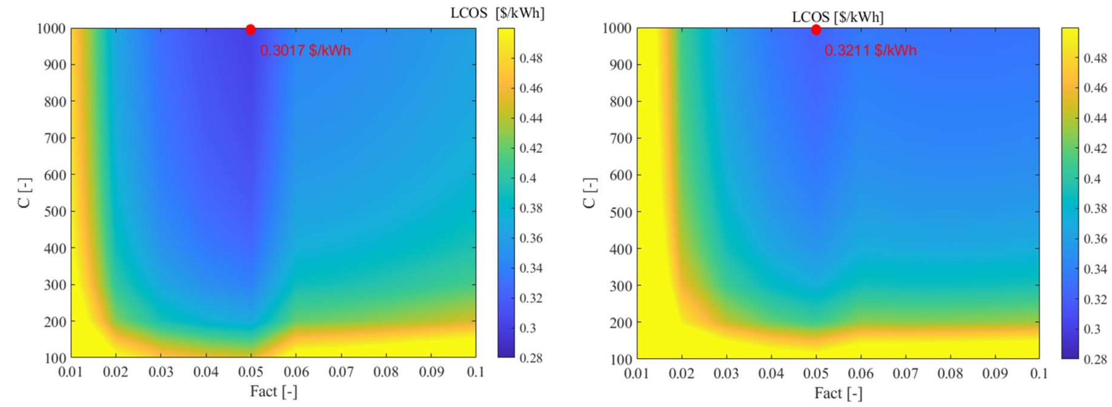

(c) (d)

Figure S18. LCOS contour plot as a function of irradiation concentration and current dilution from the PA into the EC for reversible concentrated PEC water splitting utilizing PEC design 3 (l) considering $(y_{\mathrm{PEC}}, y_{\mathrm{C}})$ =(0,0) (a), (0,1) (b), (1,0) (c), (1,1) (d).

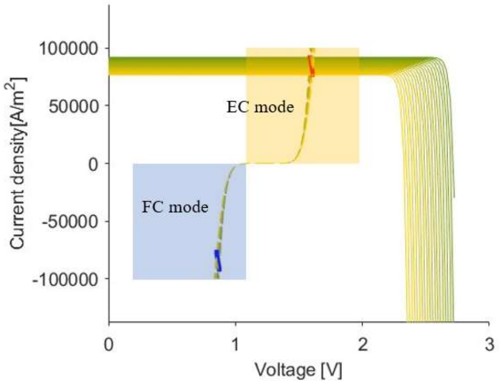

Figure S19. Yearly averaged reversible concentrated PEC design 3(l) current density voltage curves over 20 years. The red dots show the operating point for each year in EC mode (orange square). The blue dots in fuel cell mode (FC) (blue square). The FC mode operates at the same current as the EC mode. The irradiation concentration is *C*=1000. $(y_{\mathrm{PEC}}, y_{\mathrm{C}})$ =(0,0), and *Fact*=0.05.

Calculation of the cathode outlet flow rate

The CO_2 flow rate required for an average 1000 kg/day CO production rate is:

$$\dot{m}_{\mathrm{CO2}} = \frac{j_{\mathrm{op}}\, A_{\mathrm{PV}}\, M_{\mathrm{CO2}}}{2\, F_{\mathrm{a}}} \tag{S35}$$

Considering a 30% single pass conversion, the inlet flow rate is

$$\dot{m}_{\mathrm{CO2,inlet}} = \frac{\dot{m}_{\mathrm{CO2}}}{0.3} \tag{S36}$$

Considering 30% carbonation loss in the AEM, the CO_2 outlet volumetric flow rate [m^3/hour] is:

$$\dot{V}_{\mathrm{CO2,out}} = \dot{m}_{\mathrm{CO2,inlet}}(1\text{-}0.3\text{-}0.3)/\rho_{\mathrm{CO2}} \tag{S37}$$

where $\rho_{\mathrm{CO2}} = 1.98$ kg/m^3.

The CO volumetric outlet flow rate is:

$$\dot{V}_{\mathrm{CO,outlet}} = \dot{m}_{\mathrm{CO}}/\rho_{\mathrm{CO}} \tag{S38}$$

where $\rho_{\mathrm{CO}} = 1.14$ kg/m^3.

The hydrogen volumetric flow rate is:

$$\dot{V}_{\mathrm{H2,outlet}} = \frac{j_{\mathrm{op}}\, A_{\mathrm{PV}}\, M_{\mathrm{H2}}}{2\, F_{\mathrm{a}}}(1\text{-}\eta_{\mathrm{F}})\;/\rho_{\mathrm{H2}} \tag{S39}$$

where $\rho_{\mathrm{H2}} = 0.0899$ kg/m^3 and $\eta_{\mathrm{F}} = 95\%$

The total volumetric flow rate is:

$$total\ flow\ rate = \dot{V}_{\mathrm{CO2,outlet}} + \dot{V}_{\mathrm{CO,outlet}} + \dot{V}_{\mathrm{H2,outlet}} \tag{S40}$$

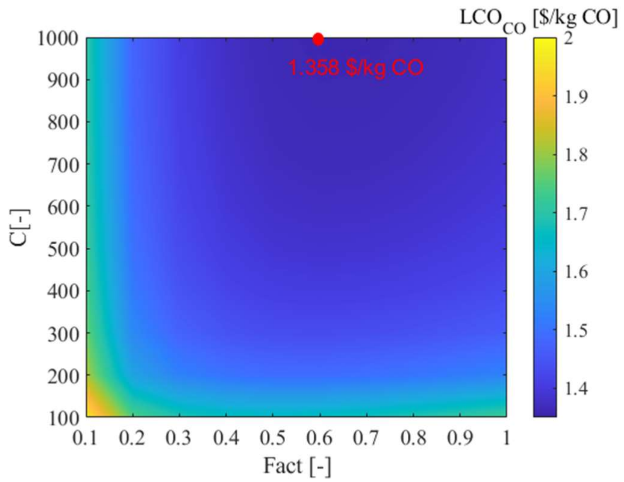

Figure S20. LCOCO contour plot as a function of irradiation concentration and current dilution from the PA into the EC for reversible concentrated PEC water splitting utilizing PEC design 3 (l) considering $(y_{\mathrm{PEC}}, y_{\mathrm{C}})$ =(1,0).

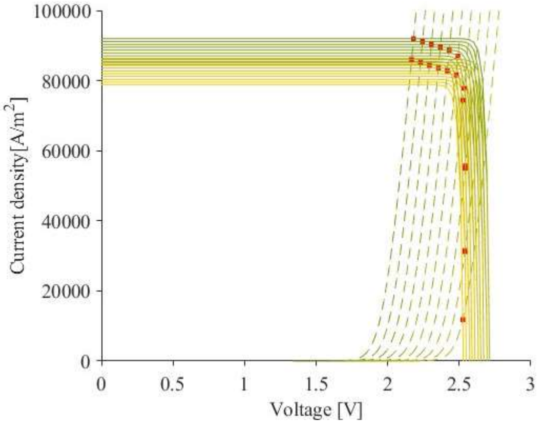

Figure S21. Yearly averaged concentrated PEC design 3(l) current density voltage curves over 20 years for CO production. The red dots show the operating point for each year. The irradiation concentration is *C*=1000. $(y_{\mathrm{PEC}}, y_{\mathrm{C}})$ =(1,0), where replacement occurs after 10 years, and *Fact*=0.6.

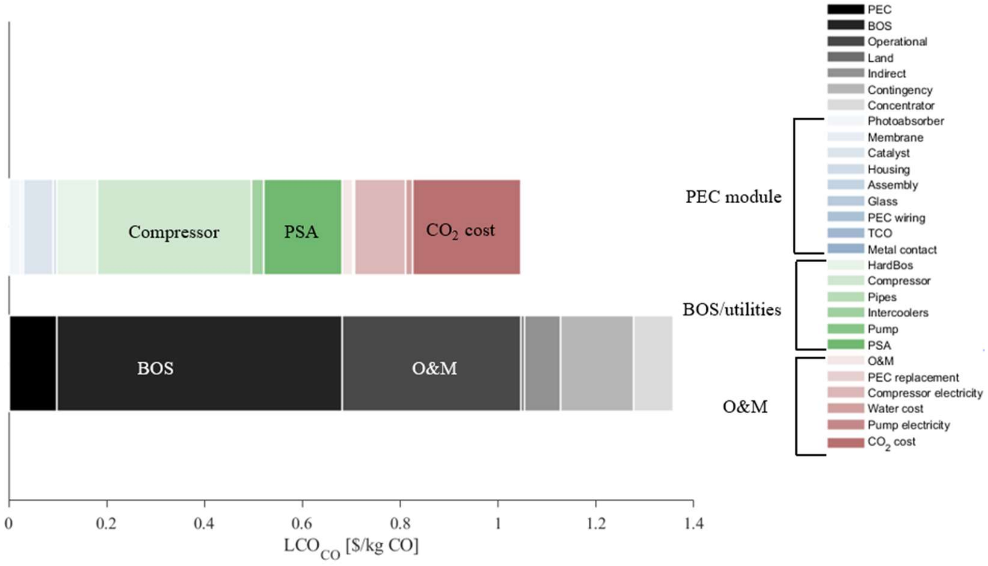

Figure S22. Current LCOCO breakdown of the concentrated PEC module design 3(l), BOS, and O&M.

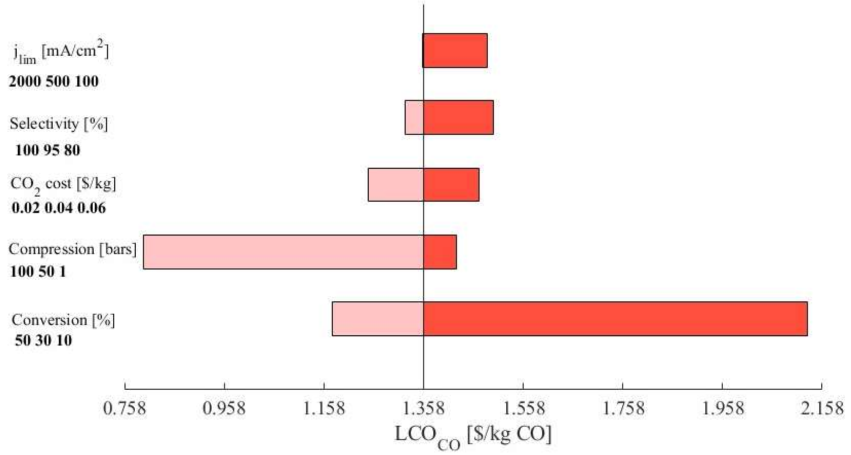

Figure S23. Sensitivity analysis for LCOCO with respect to the base case scenario (values in the middle).

S15. PEC taxonomy

Figure S24 shows the PEC taxonomy adapted from Jacobsson et al.

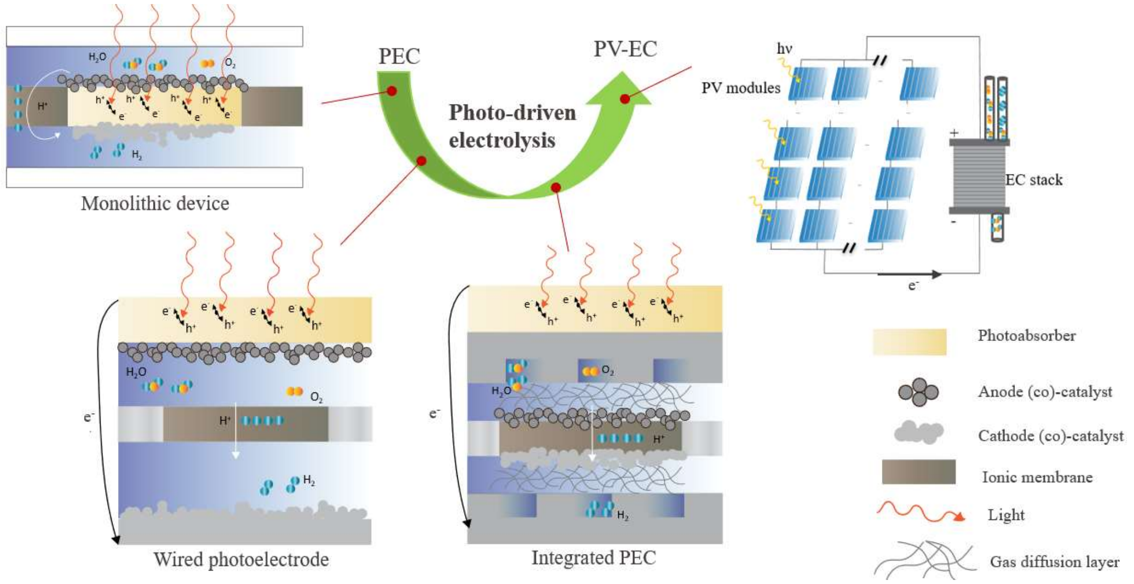

Figure S24. Solar fuel reactor taxonomy for photo-driven electrolysis.

The level of integration of the photo-absorber with the membrane-separated electrocatalyst varies across different designs, ranging from a monolithic PEC configuration to designs involving separate PV and EC components, with intermediary designs utilizing wired photoelectrodes connecting a dark cathode and an integrated PEC device. In a monolithic PEC device, the photo-absorber captures photons and converts them into electron-hole pairs. Electrons are directed towards the cathode co-catalyst, while holes are collected at the anode co-catalyst, both of which are coated onto the photo-absorber. For the water-splitting into H_2 and O_2, the anode sustains the oxygen evolution reaction and the cathode performs the hydrogen evolution reaction. The wired photoelectrode involves coating co-catalysts (here, the anode side) on one side of the photo-absorber and connecting it to a dark counter electrode (here, the cathode side). In the integrated PEC design, the photo-absorber is completely separated from the corrosive liquid electrolyte by a bipolar plate. A gas diffusion layer ensures the transport of charges and mass to the catalysts. In the PV-EC system, electron-hole pairs are generated in the PV panels,

which are physically separated from the EC stack. Electrical cables connect the PV and the EC, ensuring transport of current but also increasing ohmic, thermal, and fluidic losses.

Table S3. Design device comparison.

Device component	Design 1	Design 2	Design 3
Wired or monolithic	wired	wireless	wired
Liquid electrolyte, solid electroylte	sulfuric acid, Nafion-like membrane	sulfuric acid, Nafion-like membrane	deionized water, Nafion-like membrane
Bipolar plate	none	none	anodic and cathodic
GDL	none	none	anodic and cathodic
Catalyst coating	On both sides of the photo-absorber	On the dark side of the photo-absorber and on the dark cathode	On both sides of the ionic membrane
Photoabsorber-liquid junction	On both sides of the photo-absorber	On the dark side of the photo-absorber	None. The photo-absorber is coated on the bipolar plate

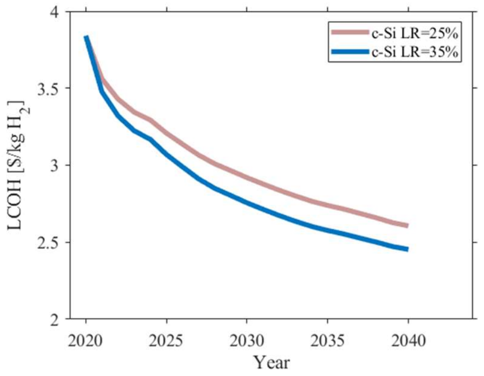

a)

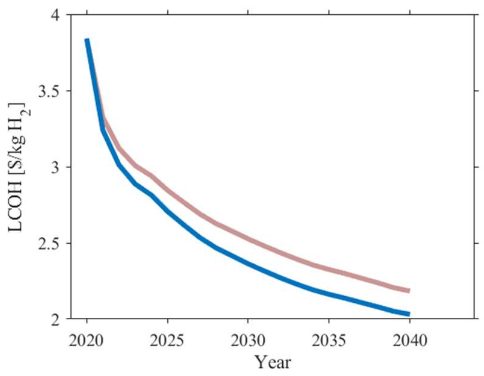

b)

Figure S25. Predicted PV-AEC cost based on a c-Si learning rate of 25% and 35% for a) 0.36 GW/year and b) 5 TW of cumulative capacity EC scenario.

S16. Local search optimization algorithm

Beginning from an initial solution, the algorithm iteratively moves to the neighbor solution.
For example, if the selected initial solution is $\{F_c = 5, F_m = 10, y_{PEC} = 0\}$, at the next iteration, the neighbor $\{F_c = 6, F_m = 10, y_{PEC} = 0\}$ is selected and the corresponding LCOF is evaluated. If the LCOF increases, the following iteration takes $\{F_c = 4, F_m = 10, y_{PEC} = 0\}$ as a solution. If the LCOF is still higher, then the initial solution was the local minimum and the next neighbor structure is selected, e.g., $\{F_c = 5, F_m = 10, y_{PEC} = 1\}$.

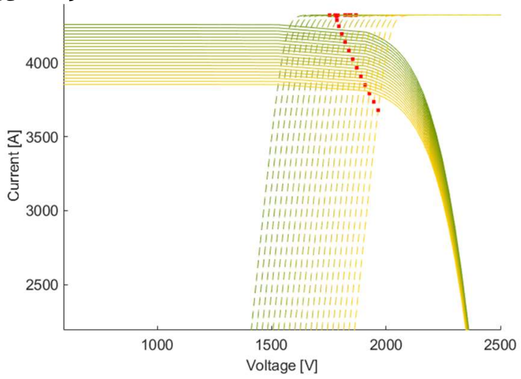

Figure S26. Yearly averaged PV-PEMEC current-voltage curves over 20 years. The red dots show the operating point for each year when utilizing a maximum power point tracker.

S17. Comparison of PEC and PV-EC systems beyond techno-economics

Table S4 summarizes additional advantages and disadvantages of PEC and PV-EC systems outside the scope of techno-economics that could benefit (niche) applications (e.g., space missions).

Pressurized systems reduce BOS costs by removing the need for a downstream compressor (on the cathode side). PV-PEMEC can operate up to 50 bars, meeting storage pressure requirements without compressors. PEC 3 has demonstrated a cathodic operating pressure of 29 bars [16], likely requiring a one-stage compressor for practical storage. AEC systems are operating below 50 bars and a one-stage compressor would be required as well. None of the PEC cells (i.e., including PEC 1 and PEC 2) have demonstrated yet operating pressure other than ambient, which therefore would currently necessitate a two-stage compressor.

PEC designs 1 and 2 have not yet shown to be operational in reverse model (i.e. fuel cell mode), although interesting approaches for uninterrupted day-night operation have been demonstrated [17]. PV-EC systems have been demonstrated as unitized regenerative fuel cells (URFCs), being able to generate heat and electricity with the same device, making them suitable for storage applications. Recently, it has also been shown for PEC 3 design that reversible (solar-driven) operation is feasible [18].

There have not yet been increased scale demonstrations for PEC designs 1 and 2, and scaling strategies are unclear. PEC design 3 has shown to be scalable, specifically when an optical concentrator is used. The photoactive area can be as high as 142.4 cm^2 (kW scale), and at the system level the solar collector area, for example, can be as high as 38.5 m^2 for one unit [16]. Above this, the combination of multiple units can be used to meet >100kW scale demands. For the PV-EC system, the PV area can be easily scaled from cm^2 to m^2 and accordingly the ECs' can be scaled from kW to MW scale at the unit scale or by combining various smaller units.

Table S4. Comparison of PEC and PV-EC systems

System	Pressure	Reversibility	Demonstrated scale	Lifetime	Versatility
**PEC 1**	Ambient	Not demonstrated	Low	Not yet competitive	Low
**PEC 2**	Ambient	Not demonstrated	Low	Not yet competitive	Low
**PEC 3**	Elevated pressure demonstrated (>30 bar)	Demonstrated [18]	Medium	Estimated to be competitive	High (with optical concentrator)
**PV-PEMEC**	Elevated pressure demonstrated (>50 bar)	Yes as unitized regenerative fuel cell	High	>10 years	Medium
**PV-AEC**	Elevated pressure demonstrated (<50 bar)	Yes as unitized regenerative fuel cell	High	>10 years	Medium

PEC designs 1 and 2 lifetime is less than 10 years and likely require frequent PEC replacement due to corrosion of the photo-electrodes. The other systems bypass a direct contact between the photo-absorbers and the electrolyte, increasing longevity. Their lifetime is estimated to be 10 to 20 years.

The versatility (i.e., ability to produce additional outputs than hydrogen and oxygen, e.g. heat and electricity) of PEC designs 1 and 2 is currently low because they have not yet demonstrated co-generation ability of heat/electricity along with hydrogen/oxygen. Generating heat at appreciable

temperature (>60°C for space applications) is unlikely to be obtained with these systems as they are not likely to work well with concentrated light and as temperature needs to be carefully managed. PEC design 3 has already demonstrated co-generation abilities (of heat/electricity) along with hydrogen/oxygen. If an optical concentrator is utilized, water convectively cools down the photo-absorber exposed to concentrated light. The oxygen stream in forward operation mode is produced with an over-stoichiometric water amount (required by its dual functionality as reactant and coolant) which is hot and this heat can be extracted and used for various heating applications. For PV-EC systems with flat PV panels, the extraction of heat is not done to not increase BOS complexity and cost.

References

1. Tembhurne, S., Haussener, S. Integrated Photo-Electrochemical Solar Fuel Generators under Concentrated Irradiation. *J. Electrochem. Soc.* **2016**, *163,* H999–H1007. DOI: 10.1149/2.0321610jes.
2. Lin, M., Haussener, S. Techno-economic modeling and optimization of solar-driven high-temperature electrolysis systems. *Sol. Energy* **2017**, *155,* 1389–1402. DOI: 10.1016/j.solener.2017.07.077.
3. Dumortier, M., Tembhurne, S., Haussener, S. Holistic design guidelines for solar hydrogen production by photo-electrochemical routes. *Energy Environ. Sci.* **2015**, *8*, 3614–3628. DOI: 10.1039/c5ee01821h.
4. de Groot, M.T., Vreman, A.W. Ohmic resistance in zero gap alkaline electrolysis with a Zirfon diaphragm. *Electrochim. Acta.* **2021**, *369,* 137684, DOI: 10.1016/j.electacta.2020.137684.
5. Nandjou, F., Haussener, S. Degradation in photoelectrochemical devices: Review with an illustrative case study. *J. Phys. D. Appl. Phys.* **2017**, *50,* 124002. DOI: 10.1088/1361-6463/aa5b11.
6. Jordan, D.C., Kurtz, S.R. Photovoltaic degradation rates-an analytical review. *Progress in photovoltaics: Research and Applications.* **2011**, *21,* 12–29. DOI: 10.1002/pip.1182.
7. Turton, R.; Bailie, R. C.; Whiting, W. B.; Shaeiwitz, J. A. Analysis, Synthesis, and Design of Chemical Processes; Pearson Education, 2008.
8. Rivera-Tinoco, R., Mansilla, C., Bouallou, C. Competitiveness of hydrogen production by High Temperature Electrolysis: Impact of the heat source and identification of key parameters to achieve low production costs. *Energy Convers. Manag.* **2010**, *51*, 2623–2634. DOI: 10.1016/j.enconman.2010.05.028.
9. *Gaseous Hydrogen Compression*, https://www.energy.gov/eere/fuelcells/gaseous-hydrogen-compression (accessed 2019-02-14).
10. Rivera-tinoco, R. M.C. Bouallou, Etude technico-économique de la production d' hydrogène à partir de l'électrolyse haute température pour différentes sources d'énergie thermique. Ph.D. Dissertation, École Nationale Supérieure des Mines de Paris 2009. https://pastel.hal.science/pastel-00005346/ (accessed 2022).
11. Rodriguez, C.A., Modestino, M.A., Psaltis, D., Moser, C. Design and cost considerations for practical solar-hydrogen generators. *Energy Environ. Sci.* **2014**, *7,* 3828–3835. DOI: 10.1039/c4ee01453g.
12. James, B.D., Baum, G.N., Perez, J., Baum, K.N. Technoeconomic Analysis of Photoelectrochemical (PEC) Hydrogen Production. *DOE report* **2009**. DOI: 10.2172/1218403. http://www.directedtechnologies.com/publications/fuel_options/PEC_Technoeconomic_Analysis-DTI.pdf.
13. J Ma, J., Man, K.L., Ting, T.O., Zhang, N., Guan, S.U., Wong, P.W.H. Approximate single-diode photovoltaic model for efficient I-V characteristics estimation. *Sci. World J.* **2013**. DOI: 10.1155/2013/230471.
14. Vartiainen, E., Breyer, C., Moser, D., Román Medina, E., Busto, C., Masson, G., Bosch, E., Jäger-Waldau, A. True Cost of Solar Hydrogen. *Sol. RRL.* **2022**, *6,* DOI: 10.1002/solr.202100487.
15. Taibi, E. et al. *Green Hydrogen Cost Reduction: Scaling up Electrolysers to Meet the 1.5 deg. C Climate Goal*; International Renewable Energy Agency, 2020.
16. Holmes-Gentle, I., Tembhurne, S., Suter, C., Haussener, S. Kilowatt-scale solar hydrogen production system using a concentrated integrated photoelectrochemical device. *Nat. Energy.* **2023**, *8*, 586–596. DOI: 10.1038/s41560-023-01247-2

17. C. Pornrungroj, V. Andrei, M. Rahaman, C. Uswachoke, H. J. Joyce, D. S. Wright, E. Reisner, Bifunctional Perovskite-$BiVO_4$ Tandem Devices for Uninterrupted Solar and Electrocatalytic Water Splitting Cycles. *Adv. Funct. Mater.* **2021**, 31, 2008182. https://doi.org/10.1002/adfm.202008182

18. Patel, M., Cattry, A., Jonin, M., Tembhurne, S., Haussener, S. Reversible photo-electrochemical device for solar hydrogen and power. *Cell Reports Physical Science* **2024**, 10.1016/j.xcrp.2024.101984.
